# Human encroachment into wildlife gut microbiomes

**DOI:** 10.1038/s42003-021-02315-7

**Published:** 2021-06-25

**Authors:** Gloria Fackelmann, Mark A. F. Gillingham, Julian Schmid, Alexander Christoph Heni, Kerstin Wilhelm, Nina Schwensow, Simone Sommer

**Affiliations:** 1 Ulm University, Institute of Evolutionary Ecology and Conservation Genomics, Ulm, Germany; 2 Smithsonian Tropical Research Institute, Panamá, República de Panamá

**Keywords:** Ecological genetics, Microbial ecology

## Abstract

In the Anthropocene, humans, domesticated animals, wildlife, and their environments are interconnected, especially as humans advance further into wildlife habitats. Wildlife gut microbiomes play a vital role in host health. Changes to wildlife gut microbiomes due to anthropogenic disturbances, such as habitat fragmentation, can disrupt natural gut microbiota homeostasis and make animals vulnerable to infections that may become zoonotic. However, it remains unclear whether the disruption to wildlife gut microbiomes is caused by habitat fragmentation per se or the combination of habitat fragmentation with additional anthropogenic disturbances, such as contact with humans, domesticated animals, invasive species, and their pathogens. Here, we show that habitat fragmentation per se does not impact the gut microbiome of a generalist rodent species native to Central America, Tome’s spiny rat *Proechimys semispinosus*, but additional anthropogenic disturbances do. Indeed, compared to protected continuous and fragmented forest landscapes that are largely untouched by other human activities, the gut microbiomes of spiny rats inhabiting human-disturbed fragmented landscapes revealed a reduced alpha diversity and a shifted and more dispersed beta diversity. Their microbiomes contained more taxa associated with domesticated animals and their potential pathogens, suggesting a shift in potential metagenome functions. On the one hand, the compositional shift could indicate a degree of gut microbial adaption known as metagenomic plasticity. On the other hand, the greater variation in community structure and reduced alpha diversity may signal a decline in beneficial microbial functions and illustrate that gut adaption may not catch up with anthropogenic disturbances, even in a generalist species with large phenotypic plasticity, with potentially harmful consequences to both wildlife and human health.

## Abstract (web summary)

Fackelmann et al. study the gut microbial composition of spiny rats across tropical forests in Panama with varying levels of protection and fragmentation in order to disentangle the relative influences of habitat fragmentation and anthropogenic disturbance on wildlife gut microbiomes. They find that habitat fragmentation on its own did not affect the gut microbiome, but the microbiomes of individuals inhabiting forest fragments affected by anthropogenic disturbance displayed a shift in community composition and structure, and were more likely to have microbiota associated with domesticated animals and their pathogens.

## Introduction

In today’s globalized world, the emergence of evermore zoonoses highlights the importance in understanding which factors facilitate the transmission of pathogens between wildlife and humans^1^. Landscape-scale disturbances that reduce habitat size and increase habitat isolation can change environmental, ecological, and host genetic factors, which play important roles in disease ecology^2^. Human activities that may lead to such a disturbance are manifold and include habitat fragmentation and isolation, whose negative impacts on wildlife health can be amplified by additional factors, such as the presence of and contact with humans^3,4^, domesticated animals^5,6^, invasive species^7^, and pathogens^7^. Generalist species are often more resilient to environmental changes, important pathogen reservoirs, and sources of zoonotic diseases^8,9^. Examining their ability to alter their gut microbial composition and its genes, known as metagenomic plasticity, when faced with human-driven environmental changes could help to understand the dynamics of emerging diseases from wildlife.

While adaptation is most commonly associated with host genomics, the adaptive potential of the gut microbiome remains understudied^10^. The gut microbiome is an integral part of an animal’s well-being, as the microbial community provides essential nutritional services and protection against gut-invading pathogens to its host and is an important driver of mucosal immunity maintaining gut homeostasis^11,12^. Consequently, shifts in this symbiont’s diversity pattern beyond the normal range of variation is linked to the health of its host^13–15^. These shifts can be adaptive, indicating metagenomic plasticity, as well as maladaptive if they are associated with a decrease in beneficial functions, an increase in pathogens causing disease, or a decline in fitness^10,16^. The latter is often referred to as dysbiosis, though a more detailed definition of the patterns of dysbiosis is lacking^17^.

In the Anthropocene, habitat fragmentation and other anthropogenic disturbances threaten wildlife^18^. Their effects on the gut microbiome have been studied together^19–22^, but there is a gap in understanding if habitat fragmentation per se perturbs the gut microbiome or if the amplification by additional anthropogenic factors, such as contact with humans, domesticated animals, invasive species, and pathogens poses a greater threat to wildlife gut microbial health. Here, we studied the gut microbial composition of 384 individuals of a generalist rodent species, Tome’s spiny rat *Proechimys semispinosus*, in seventeen study sites in three tropical landscapes differing in their degree of anthropogenic disturbance in Panama, Central America. The three landscapes encompassed: (1) protected continuous tropical forests and (2) protected forested islands in the Panama Canal that allow us to study the effects of fragmentation on its own—both landscapes are largely undisturbed by human activities—and (3) nearby unprotected forested fragments embedded in an agricultural matrix that are subjected to further anthropogenic disturbances in addition to habitat fragmentation. By comparing both protected landscapes to heavily human-disturbed, fragmented sites, our unique study design allowed us to, first, pick apart the effects of habitat fragmentation (i.e., habitat reduction and isolation) from those of additional anthropogenic disturbances (i.e., contact with humans, domesticated animals, invasive species, and pathogens within an agricultural matrix) on the gut microbiome and, second, to meticulously characterize the changes in gut community composition and metagenomic functional potential. Our results show that habitat fragmentation on its own does not impact the gut microbiome of our generalist study species. However, the gut microbiomes of individuals inhabiting forest fragments embedded in an agricultural matrix with additional anthropogenic disturbances had lower alpha diversity, displayed a shift in community composition and greater dispersion (i.e., increased heterogeneity) in gut microbial community structure between individuals, along with a shift in potential metagenome functions. In addition, we found that taxa associated with both domesticated animals (and their pathogens) were over-represented in these microbiomes. These findings could be early warning signs of the gut microbiome’s loss of resilience^23^. Considering its integral role in host health, such a loss could not only be detrimental to wildlife health, but could also promote microbial pathogens with zoonotic potential.

## Results

### Additional anthropogenic disturbance but not habitat fragmentation per se lowers gut bacterial diversity within individuals

**Fig. 1:**
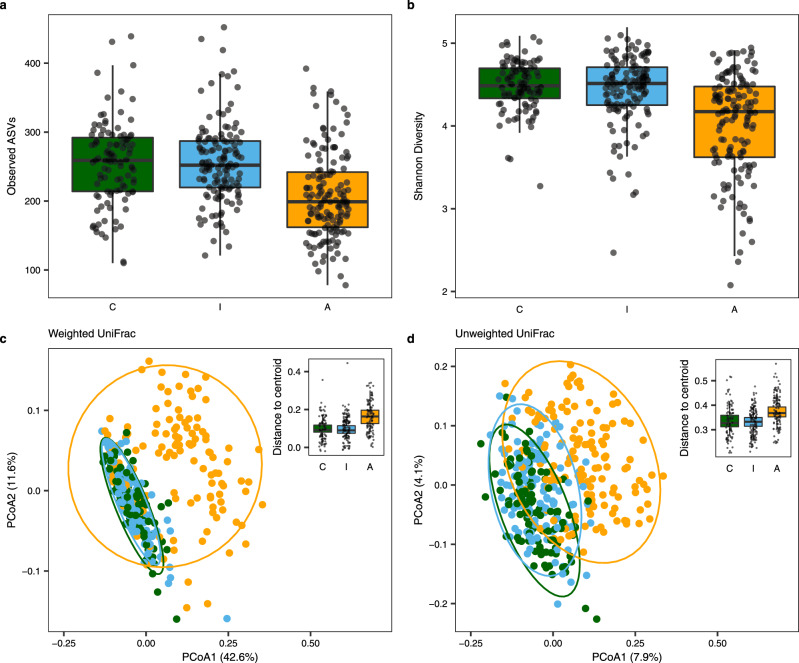
Additional anthropogenic disturbance influences gut microbial diversity and structure in the generalist spiny rat *P. semispinosus*. **a**, **b** Gut microbial alpha diversity within spiny rat individuals inhabiting protected, continuous forests (control landscape C, green, *n* = 103 individuals), protected, isolated forest fragments (islands) with no further anthropogenic disturbance (landscape I, blue, *n* = 136 individuals), and forest fragments embedded in an agricultural matrix with additional anthropogenic disturbance (landscape A, orange, *n* = 145 individuals). Alpha diversity is measured by **a** the observed number of ASVs (i.e., ASV diversity) and **b** Shannon diversity, which also accounts for species abundance. Boxplots display the median with the center line, hinges represent 25th and 75th percentiles, and whiskers extend to 1.5 times the interquartile range. Each dot represents a sampled spiny rat individual. **c**, **d** Principal coordinates analysis (PCoA) plots of the diversity between spiny rat individuals accounting for phylogenetic diversity and weighted (**c**) and unweighted (**d**) for abundance using UniFrac distances. Each dot represents a *P. semispinosus* individual sampled in landscape C (green), landscape I (blue), or landscape A (orange). Sample sizes in the PCoA plots equal to **a** and **b**. Ellipses indicate 95% confidence intervals. Inset boxplots show the weighted (**c**) and unweighted (**d**) UniFrac distances to the study site centroids within each landscape (*n* = 89 individuals in C, *n* = 126 individuals in I, *n* = 107 individuals in A). Boxplot parameters the same as in **a** and **b**.

To test if habitat fragmentation per se or additional anthropogenic disturbance impacts the gut microbiome of a generalist species (Tome’s spiny rat) inhabiting lowland tropical rainforests in Central America (Supplementary Figs. 1 and 2), we first calculated the diversity of microbes within each of the 384 sampled individuals (i.e., the alpha diversity) using three metrics: the observed number of amplicon sequence variants (ASVs), Shannon diversity, and Faith’s phylogenetic diversity (PD). We used generalized linear mixed models, which allowed us to control for the lack of independence due to site-specific effects within landscapes and extraction batches. Using model selection based on the information-theoretic (IT) approach^24^, we found very strong support for an effect of landscape on all three alpha diversity metrics: observed number of ASVs (ΔAIC_C_ = 19.63, R^2^_GLMM(m)_ = 0.473, R^2^_GLMM(c)_ = 0.579, Fig. 1a, Supplementary Data 1); Shannon diversity, in which alpha diversity is weighted for abundance (ΔAIC_C_ = 5.84, R^2^_GLMM(m)_ = 0.150, R^2^_GLMM(c)_ = 0.171, Fig. 1b, Supplementary Data 2); and Faith’s PD, which controls for phylogenetic relatedness (ΔAIC_C_ = 6.05, R^2^_GLMM(m)_ = 0.438, R^2^_GLMM(c)_ = 0.480, Supplementary Fig. 3, Supplementary Data 3). For all three alpha diversity metrics, this landscape effect was driven by a lower alpha diversity in forest fragments surrounded by an agricultural matrix (landscape A, subjected to additional anthropogenic disturbance) than in either protected continuous forests (landscape C, our control) or protected forested islands (landscape I, subjected to fragmentation without further anthropogenic disturbance, Supplementary Fig. 2) (observed number of ASVs: *β*_C_ = 242.4 (95% CI = 211.3–278.1), *β*_I_ = 247.9 (95% CI = 203.6–301.9), *β*_A_ = 202.4 (95% CI = 165.8–246.9); Shannon diversity: *β*_C_ = 4.453 (95% CI = 4.260–4.655), *β*_I_ = 4.417 (95% CI = 4.054–4.813), *β*_A_ = 4.053 (95% CI = 3.3707–4.430; Faith’s PD: *β*_C_ = 21.61 (95% CI = 19.03–24.55), *β*_I_ = 21.88 (95% CI = 18.15–26.38), *β*_A_ = 19.33 (95% CI = 15.99–23.36); Supplementary Data 4). Because lower alpha diversity was observed in human-disturbed, fragmented landscapes (A), but in neither of the protected landscapes with (I) and without (C) habitat fragmentation, these results indicate that habitat fragmentation per se does not impact gut bacterial diversity within individuals, but that its combination with additional anthropogenic disturbance does. Furthermore, the results suggest that this effect may be driven more by rare taxa than abundant ones, since the effect was less pronounced when accounting for abundance. The effects of field season and sex were poorly supported by AIC_C_ model comparison for any of the three alpha diversity metrics (Supplementary Data 1–3). In addition, in all three alpha diversity models, spatial autocorrelation between capture sites was poorly supported by AIC_C_ model comparison (Supplementary Data 5) and, because models including this parameter were less parsimonious than their counterparts without the parameter, we opted against including this parameter in our final alpha diversity models.

### Additional anthropogenic disturbance but not habitat fragmentation per se causes both compositional shifts and greater dispersion of gut bacterial community structures

Next, we examined if and how habitat fragmentation and additional anthropogenic disturbances impact gut microbial diversity between individuals, known as beta diversity. We first tested for shifts in gut community composition by using the permutational multivariate analysis of variance (PERMANOVA) test, which tests if the centroids of all groups are the same. To account for the lack of independence between study sites and landscapes, study site was nested within landscape and passed through the ‘strata’ argument of the *adonis* function in the vegan package^25^. However, because the ‘strata’ argument impacts *p*-values but not the calculation of AIC_C_, we report results from both null hypothesis significance testing and IT model selection. In addition, we report Cohen’s *d* effect sizes and 95% confidence intervals^26,27^ calculated using coordinates from the first two PCoA axes^28^. Again, using IT model selection, we found strong support for an effect of landscape type on compositional shifts in beta diversity with regards to both weighted (ΔAIC_C_ = 53.29, PCoA axis 1: Cohen’s *d*_A-C_ = −1.053 (95% CI = −1.332 to −0.771), Cohen’s *d*_A-I_ = −1.110 (95% CI = −1.375 to −0.841), Cohen’s *d*_C-I_ = −0.030 (95% CI = −0.286 to 0.227); PCoA axis 2: Cohen’s *d*_A-C_ = −0.904 (95% CI = −1.176 to −0.629), Cohen’s *d*_A-I_ = −0.847 (95% CI = −1.100 to −0.593), Cohen’s *d*_C-I_ = 0.109 (95% CI = −0.148 to 0.365); Fig. 1c, Supplementary Data 6) and unweighted UniFrac distance metrics (ΔAIC_C_ = 21.74, PCoA axis 1: Cohen’s *d*_A-C_ = −1.501 (95% CI = −1.805 to −1.193), Cohen’s *d*_A-I_ = −1.501 (95% CI = −1.790 to −1.208), Cohen’s *d*_C-I_ = 0.058 (95% CI = −0.198 to 0.314); PCoA axis 2: Cohen’s *d*_A-C_ = −1.427 (95% CI = −1.428 to −0.857), Cohen’s *d*_A-I_ = −0.684 (95% CI = −0.930 to −0.436), Cohen’s *d*_C-I_ = 0.499 (95% CI = 0.233 to 0.763); Fig. 1d, Supplementary Data 7). This effect of landscape is also supported by null hypothesis significance testing (weighted UniFrac: *p* < 0.001 for 9,999 permutations, R^2^ = 0.142, F = 37.70; unweighted UniFrac: *p* < 0.001 for 9,999 permutations, R^2^ = 0.052, F = 11.37; Supplementary Data 8). Although both metrics account for phylogenetic relatedness, weighted UniFrac takes ASV abundance into consideration as well. These results show that the gut bacterial community composition of spiny rats inhabiting the disturbed forest fragments embedded in an agricultural matrix was shifted in comparison to the microbial community of spiny rats inhabiting protected landscapes with (I) and without (C) habitat fragmentation, which themselves had very similar microbial compositions (Fig. 1c and d, Supplementary Fig. 4). These results demonstrate that fragmented landscapes with additional anthropogenic disturbance (e.g., contact to domesticated animals, invasive species, humans, and their pathogens) can alter gut microbial community structures. This shift is largely driven by changes in the most abundant gut microbes, rather than differences in their presence or absence, because the effect was strongest when accounting for abundance.

Next, we assessed if—in addition to shifting gut microbial community composition—habitat fragmentation combined with additional anthropogenic disturbance also impacted gut microbial community dispersion by testing for differences in homogeneity of variance between the landscapes using the distances calculated by PERMDISP2^29^. We found strong support for differences in dispersion between the landscapes for both weighted (ΔAIC_C_ = 11.49, R^2^_GLMM(m)_ = 0.339, R^2^_GLMM(c)_ = 0.350, Fig. 1c insert, Supplementary Data 9) and unweighted UniFrac distances (ΔAIC_C_ = 4.10, R^2^_GLMM(m)_ = 0.251, R^2^_GLMM(c)_ = 0.271, Fig. 1d insert, Supplementary Data 10), while accounting for study site-specific effects (see Methods). These differences in variance were driven by the forest fragments embedded in an agricultural matrix, which had greater dispersion in beta diversity than study sites in the continuous forests or on the forested islands (weighted UniFrac: *β*_C_ = 0.107 (95% CI = 0.093–0.122), *β*_I_ = 0.104 (95% CI = 0.082–0.132), *β*_A_ = 0.178 (95% CI = 0.138–0.230); unweighted UniFrac: *β*_C_ = 0.338 (95% CI = 0.326–0.350), *β*_I_ = 0.335 (95% CI = 0.310–0.361), *β*_A_ = 0.379 (95% CI = 0.347–0.414); Supplementary Data 11). These findings reveal that it is not the effect of habitat fragmentation per se, but fragmentation in combination with additional anthropogenic disturbance that causes greater dispersion in community structure between individuals. This suggests that human disturbances at the landscape level can both shift gut microbial community structure and increase community dispersion in wildlife gut microbiomes, even in generalist species that are considered to be more resilient to environmental changes^9^. In addition, the most abundant gut microbes, as opposed to more rare taxa, had a stronger effect on these patterns of gut community change. The effects of field season and sex for both PERMANOVA and PERMDISP2 analyses were poorly supported by AIC_C_ model comparison, regardless of the UniFrac metric used (Supplementary Data 7-10). In addition, in all PERMDISP2 models, spatial autocorrelation between capture sites was poorly supported by AIC_C_ model comparison (Supplementary Data 12) and, because models including this parameter were less parsimonious than their counterparts without the parameter, we opted against including this parameter in our final PERMDISP2 models.

### Additional anthropogenic disturbance but not habitat fragmentation per se causes shifts in gut microbial community structure and metagenome functions

**Fig. 2:**
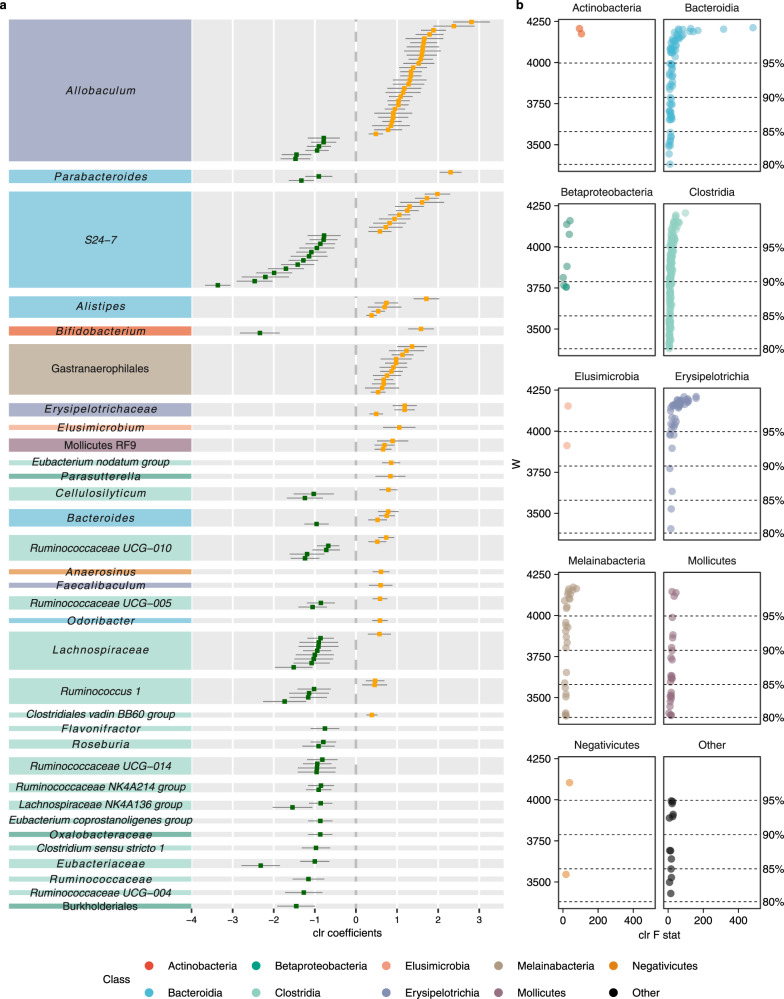
Differentially abundant ASVs between the protected landscapes (C and I) and fragmented landscape A with additional anthropogenic disturbance identified by ANCOM. **a** The 142 ASVs out of a total of 4213 ASVs determined to be differentially abundant (at *w* > 95%) between landscapes C and I (green) and landscape A (orange). Each dot is an ASV plotted in decreasing order by its coefficient with confidence intervals as 1.96 times the standard error. Dots to the right of center zero in orange represent ASVs over-represented in landscape A, while dots to the left in green represent ASVs over-represented in landscapes C and I. ASVs are grouped by most detailed taxonomic assignment. Taxa are colored according to class. **b** Volcano plots of differentially abundant ASVs (at *w* > 80%) and those leading up to the determined cutoff value *w*_*0*_ = 0.95 are displayed in **a**. Each dot represents an ASV plotted by its F statistic as a measure of effect size and by its W value, which is a count of how many times the null hypothesis was rejected for that particular ASV. The dots are colored by taxonomic class as in **a**.

We conducted an in-depth analysis of compositional differential abundance to determine which taxa were driving the differences in beta diversity between the forest fragments (A) and both protected landscapes with (I) and without (C) fragmentation (landscapes I and C were pooled together since the results from both the PERMANOVA and PERMDISP2 tests showed that there were no statistically supported differences in beta diversity between these landscapes). To do so, we applied an analysis of composition of microbiomes (ANCOM) test^30^ and found 142 ASVs across 33 taxa and nine classes to be differentially abundant (at *w* > 95%, see Methods) between the study sites in the fragmented forest landscape and those in the protected continuous forests and forested islands (Fig. 2). The taxonomic group with the greatest number of differential ASVs was *Allobaculum* (34 ASVs, class Erysipelotrichia), followed by the *S24-7* family (23 ASVs, class Bacteroidia), and the Gastranaerophilales order (12 ASVs, class Melainabacteria) (Fig. 2a). On the one hand, several taxa containing differentially abundant ASVs were over-represented in the protected landscapes without additional anthropogenic disturbance (C and I), such as *Clostridium sensu strincto 1*, *Roseburia*, and the *Eubacterium coprostanoligenes* group. On the other hand, some ASVs belonged to taxa that were over-represented in the fragmented landscape with additional anthropogenic disturbance (A), such as *Odoribacter*, Gastranaerophilales, and members of Mollicutes RF9. While these and 17 other taxa were over-represented either in landscapes C and I or in landscape A, the remaining ten taxonomic groups did not show such a distinct division and instead contained some ASVs that were more abundant in landscapes C and I, while other ASVs were more abundant in landscape A, most notably *Allobaculum* and the *S24-7* family. More details are presented in the Supplementary Information. These results suggest that the patterns of gut microbial community change observed here may be driven by shifts in abundances of taxa already present in the gut microbiome (Supplementary Fig. 5), along with both the loss of certain (potentially commensal) species and the gain of other (potentially pathogenic) species.

To better understand the shifts in genomic functional potential between the microbiomes of spiny rats inhabiting protected versus fragmented and anthropogenically disturbed landscapes, we used PICRUSt2^31–34^ in conjunction with ANCOM^30^. Four predicted pathways were determined to be differentially abundant between the protected landscapes (C and I) and the fragmented landscape with additional anthropogenic disturbance (A; at *w* > 95%, see Methods): pathway 1861 (formaldehyde assimilation II—RuMP Cycle; Supplementary Fig. 6), pathway 2941 (L-lysine biosynthesis II; Supplementary Fig. 7), the teichoic acid pathway (teichoic acid (poly-glycerol) biosynthesis; Supplementary Fig. 8), and pathway 7210 (pyrimidine deoxyribonucleotides biosynthesis from CTP; Supplementary Fig. 9). Three of the four pathways were predominately more abundant in landscape A (pathway 1861, pathway 2941, and the teichoic acid pathway; Supplementary Figs. 6–8), while pathway 7210 was predominately more abundant in landscapes C and I (Supplementary Fig. 9). More details are presented in the Supplementary Information. The taxa associated with the differentially abundant predicted pathways were the same as those belonging to the differentially abundant ASVs identified in the previous paragraph, with the exception of the additional taxa associated with pathway 7210 (Supplementary Information; compare Fig. 2 to Supplementary Figs. 6–9). Although these four predicted pathways were predominantly more abundant in either landscape C and I or in landscape A, there was no distinct pattern in which one particular pathway was always more abundant in a certain landscape across all the taxa containing that pathway, thus reflecting again the results based on differentially abundant ASVs (compare Fig. 2a to Supplementary Figs. 6a–9a). Altogether, this demonstrates that habitat fragmentation per se does not alter the gut microbial functional potential, but that habitat fragmentation in combination with additional anthropogenic disturbance does. In addition, the changes in metagenome functions seem to be driven by shifts in the predicted abundances of pathways already present in the gut metagenome.

## Discussion

Faced with global change in the Anthropocene, wildlife all over the world has come under threat as humans advance further into wildlife habitats^35,36^. Of increasing interest in conservation biology is to understand the evolutionary responses of wildlife to human-driven selection pressures^18^. Within this context, it has been proposed that, although species may adapt over time under selection of their genes, gut microbiomes may carry potent adaptive potential due to the short generation times of microbes, the possibility to transfer genes, and because of their sheer size in numbers, meaning evolution can occur at a faster rate^10^. In this study, we sequenced the gut microbiomes of 384 Tome’s spiny rat *P. semispinosus* individuals inhabiting three landscapes that differed in their degree of habitat fragmentation and further anthropogenic disturbance. Our results show that the gut microbiomes of spiny rat individuals inhabiting fragmented landscapes with additional anthropogenic disturbance (such as contact to domesticated animals, invasive species, humans, and their pathogens) had lower alpha diversity and a shifted and more dispersed gut bacterial community composition, indicating that human disturbances at the landscape level can have both location and dispersion effects on the gut microbiomes of wildlife, even in generalist species that are considered to be more resilient to environmental changes^9^. Habitat fragmentation alone, however, had no supported effects on the gut microbiome of this generalist rodent.

In this study, we observed two distinct patterns of gut microbial community change in spiny rats inhabiting fragmented landscapes with additional anthropogenic disturbance that were not observed in spiny rats inhabiting protected landscapes without additional human impact: gut microbial compositional shifts and greater dispersion in beta diversity. The latter aligns with the observations made by several studies that the microbial composition of hosts with perturbed microbiomes often displays greater heterogeneity of variance or greater dispersion, meaning there are greater changes in microbial structure between individuals^14,15,37–43^. This has been termed the Anna Karenina Principle, derived from Leo Tolstoy’s famous novel of the same name, which states that all healthy microbiomes are alike, while perturbed microbiomes are all different^41^. This pattern of dispersion in the face of a stressor may be caused by stochastic i.e., random processes^41^. Such processes could be driven by the random colonization of bacteria novel to the individual’s gut microbiome in the anthropogenically more disturbed landscape or by random extinction of resident bacteria in the face of novel competition caused by some spiny rat individuals that come into more intense contact with human activities than others, perhaps due to the proximity of their home range to the edges between the forest patches and the agricultural matrix humans live in. This could cause a stochastic drift in the structural composition of the gut microbiome, leading to a dispersed pattern in beta diversity^44^.

Greater variation in beta diversity coupled with a reduction in alpha diversity could be early warning signs of declining host health^23,41^. Gut microbial maladaptation has been linked to a wide range of diseases (ref. ^16^ and references herein) and infections^14,45^ and may facilitate co-infections^15^. Wildlife health and susceptibility to pathogens play important roles in the Anthropocene, as humans come into increasing contact with potentially maladaptive wildlife, which could lead to the transmission of zoonotic diseases.

Community structure can be molded not only by stochastic processes but also by deterministic ones (such as habitat specialization or environmental filtering^46^; for an in-depth discussion on the influence of stochastic versus deterministic effects on beta diversity, see ref. ^47^), which may result in heterogeneous selection depending on the environment^48,49^. Inhabiting the landscape that is not only affected by fragmentation but also by further anthropogenic disturbance—surrounded by a matrix composed of patchworks of agricultural fields, various domesticated animals, roads, and houses—could mean that individuals are subjected to a heterogeneous palette of microbes, wherein each individual is exposed to a unique composition of microbes. This can lead to a dispersed pattern in beta diversity, driven by inter- and intraspecific competition among gut microbes together with the effects of host mucosal immunity, which determine an individual’s microbiome composition. This would be an example of heterogeneous selection^49^. We found a wide range of ASVs belonging to taxa associated with domesticated animals to be over-represented in the gut microbiomes of spiny rats in anthropogenically disturbed landscapes. For example, members of the bacterial class Mollicutes contain a wide range of agriculturally relevant pathogens, such as *Mycoplasma bovis*, a causative agent of pneumonia and mastitis in cattle^50^, *M. gallisepticum*, a consequential pathogen in poultry that causes chronic respiratory disease in chickens and turkeys^51^, and *M. suis*, which causes anemia in pigs^52^. In addition, hemotrophic mycoplasma are known to infect dogs and cats^53^, while *Odoribacter*—isolated from the oral microbiomes of dogs and cats—causes periodontitis in infected animals^54^, and the family *Erysipelotrichaceae* has been linked to gut inflammation in mice models and metabolic disorders in hamster models^55^. However, these higher-ranking taxa do not only include pathogens and since our results are based on 16S rRNA gene sequencing, which rarely permits reliable taxa identification at the species level, further investigations are required.

The shift in gut microbial community composition in spiny rats inhabiting fragmented landscapes with additional anthropogenic disturbance can be seen as further evidence of an adaptive shift in microbiome composition^41,49^. We found that some differentially abundant taxa, although present across all landscapes, showed a shift in their abundance towards either the two protected landscapes or the anthropogenically more disturbed landscape. This supports the notion of the up- or downregulation of existing taxa in response to stressors, more than the acquisition of novel or loss of established microbial members. Among these taxa is the family *S24-7*, which is able to ferment a wide range of carbohydrates, leading to the conclusion that it may strive in several different niches in the gut environment^56^. In addition, the genus *Allobaculum* has demonstrated rapid acclimation to changes in sugar availability by being able to quickly assimilate added glucose, enabling it to take over cultured microbiomes^57^. *A. stercoricanis* is the only species within the genus *Allobaculum* and is not only a common member of the gut microbiome of dogs, but also produces the short chain fatty acid (SCFA) butyrate as a byproduct of fermentation^58^. Among other functions, SCFAs play pivotal roles in maintaining gut health^59^. Several of the ASVs that were over-represented in the protected landscapes belong to taxa known for their production of short chain fatty acids, such as *Clostridium sensu strincto 1*, *Roseburia*, and the *Eubacterium coprostanoligenes* group^60–63^. Taken together, this suggests a potential for gut microbial adaptability, which could translate into increased metagenomic plasticity for this rodent host faced with the selective pressures of anthropogenic disturbance.

Based on predicted pathway abundances, spiny rats show potential for gut microbial metagenomic plasticity in response to a more intense anthropogenic disturbance (but not habitat fragmentation per se). We found four predicted pathways that were differentially abundant between landscape A and landscapes C and I. The pathway over-represented in the protected landscapes (pathway 7210: pyrimidine deoxyribonucleotides biosynthesis from CTP) represents a salvaging pathway of free bases and nucleosides to biosynthesize pyrimidine nucleotides, which can be preferable to *de novo* biosynthesis pathways that require more energy^64,65^. In contrast, the pathways over-represented in the fragmented landscape with additional anthropogenic disturbance (pathway 1861: formaldehyde assimilation II—RuMP Cycle; pathway 2941: L-lysine biosynthesis II; and the teichoic acid pathway: teichoic acid (poly-glycerol) biosynthesis) encompass the synthesis of cell wall components^65–69^, which play a key role in pathogenesis^67,70^, and the assimilation of formaldehyde^71^ generated during inflammation^72^ and during the oxidation of methane^65,71^ (emitted in noninsignificant amounts by ruminant livestock and their manure^73^). More details regarding the pathway functions are presented in the Supplementary Information. This could indicate selective pressures stemming from living in the vicinity of methane-emitting livestock or from gut inflammation in the spiny rats and a need for greater fortification of cell walls, perhaps to enhance pathogen recognition. However, more experimental evidence would be needed to demonstrate this and it should be noted that the potential metagenomic functions of the gut microbes in these spiny rats are derived from predicted pathways based on short 16S rRNA amplicon sequencing. Furthermore, the accuracy of these metagenomes is dependent on the information annotated in databases, which do not encompass the full range of possible bacterial functional capacity, especially for lesser-known and understudied bacterial taxa. All four differentially abundant pathways were present in spiny rat microbiomes across all the landscapes, as opposed to certain pathways being present exclusively in one landscape type. This supports the notion of the up- or downregulation of existing pathways in response to stressors, as was the case with the differentially abundant microbes that, to a greater extent, reflected a shift in their composition and, to a lesser extent, a gain or loss of specific bacterial taxa.

In conclusion, although our results suggest that the gut microbiome of the generalist spiny rat may possess the metagenomic plasticity to adapt to anthropogenic disturbances by shifting its gut microbial composition and functional potential, the increased variation and dispersion in gut microbial structure following the Anna Karenina Principle suggest that this adaptation may not be occurring fast enough. As human encroachment into wildlife habitats increases, it remains to be seen if this capacity for gut microbial adaptation can keep up with stacking anthropogenic disturbances. It also highlights the urgency for further studies to assess the generality of our results across a broader range of mammalian species and geographic locations. While the gut microbiome is a key component in host health and likely plays a role in (wildlife) susceptibility to pathogens, the direct mechanisms driving the gut microbiome’s potential role in the transfer of zoonotic diseases to humans remains to be elucidated.

## Methods

### Study area and sample collection

This study was conducted in the Panama Canal area, Panama, Central America (Supplementary Fig. 1), a unique study area which allowed us to distinguish between the effects of habitat fragmentation per se (i.e., habitat reduction and isolation) from those of additional anthropogenic disturbance (i.e., contact with humans, domesticated animals, invasive species, and their pathogens within an agricultural matrix). The construction of the Panama Canal over a century ago led to the flooding of the surrounding areas, resulting in the isolation of mountain tops that became isolated, forested islands, which were placed under protection that continues to this day. The Panama Canal is bordered by similar tropical lowland rainforests (Barro Colorado Natural Monument) that are also protected. Anthropogenic disturbance by agriculture and increased human settlements in the north-east of the study area begins 25 km away from the protected forests^74^. The result is a unique arrangement of three different landscapes: (1) continuous tropical forests that are largely undisturbed anthropogenically due to their protected status (continuous forest, control group, C); (2) tropical forest fragments in the form of forested islands surrounded by water (i.e., forest fragments embedded in a water matrix) that are also protected and thus not subjected to additional anthropogenic disturbance (forested islands, I), allowing us to study the effects of fragmentation alone; and (3) unprotected fragmented tropical forests embedded in an agricultural matrix (forested fragments in an agricultural matrix, A) that are subjected to anthropogenic disturbances in addition to fragmentation (Supplementary Fig. 2). This setup allowed for the categorization of these landscapes based on the matrices in which the forest patches are embedded. For the continuous forests (landscape C), the protected forest “patches” are embedded in a forest matrix, meaning there are no patches per se, but rather the forest is continuous, therefore this landscape is our control group. Although the islands (I) also harbor protected forest patches like landscape C, these patches are embedded in a matrix of water, since the islands are located in the Panama Canal, allowing the study of fragmentation alone and not fragmentation in combination with additional anthropogenic disturbances. Finally, the forest fragments (landscape A) are unprotected forest patches embedded in an agricultural matrix subjected to additional anthropogenic disturbance such as contact with humans, domesticated animals, invasive species, and pathogens. The forest patches in all three landscapes are tropical lowland rainforests. The difference between the landscapes lies in the matrices, which are distinctly different (forest, water, and agriculture) and thus allow the landscapes to be categorized into three groups (C, I, and A; Supplementary Fig. 2).

For analysis of the gut microbiome, we sampled one of the most abundant terrestrial mammal species common to all three landscapes, the generalist Tome’s spiny rat *Proechimys semispinosus*^75^. This rather large rodent species, whose weight can exceed 700 g^76^, primarily feeds on fruits and seeds and is thus an important seed disperser in the tropics^77,78^. Tome’s spiny rat is solitary, though not territorial, meaning home-ranges between individuals can overlap and individuals may share or co-occupy burrows^79^. Similar to generalist rodents in temperate forests and habitat generalists overall, this rodent species is able to adapt to and exploit heterogeneity in its environment by, for example, adjusting its reproductive efforts and output^80^. Within each landscape, rats were sampled in at least five different study sites, all at a similar altitude near the Panama Canal (sites C1–C5 *n*_*C*_ = 103, I1–I6 *n*_*I*_ = 136, and A1–A6 *n*_*C*_ = 145, Supplementary Fig. 1). Spiny rat individuals were live-trapped and fecal samples collected as described in detail in ref. ^75^. during three field seasons (October 2013 to May 2014, October 2014 to May 2015, and September 2016 to April 2017). In the field, fecal samples were stored in RNAlater and transferred to −20 °C upon daily return back to the field station.

This study was carried out within the framework of the German Science Foundation (DFG) Priority Program SPP 1596/2 Ecology and Species Barriers in Emerging Infectious Diseases (SO 428/9-1, 9-2, with full ethical approval according to the Smithsonian IACUC protocol 2013-0401-2016-A1-A7 and 2016-0627-2019-A1-A2). Permission to export samples to Germany was granted by the Panamanian government (SE/A-21-14, SE/A-69-14, SEX/A-22-15, SEX/A-24-17, SEX/A-120-16, and SEX/A-52-17).

### DNA extraction, PCR amplification, library preparation, and 16S rRNA gene sequencing

DNA was extracted from fecal samples using the NucleoSpin Soil Kit (Macherey-Nagel, Germany) following the manufacturer’s instructions. This protocol includes a bead-beating step to mechanically lyse bacterial cells using ceramic beads that was carried out using the SpeedMill PLUS (Analytik Jena, Germany) with two 3-minute cycles of bead-beating separated by a 3-minute resting period. Following the manufacturer’s instructions, the supernatant was transferred from the tubes with ceramic beads to new collection tubes following centrifugation and prior to precipitation. The remaining steps were conducted according to the protocol instructions. Twelve extraction blanks containing only the extraction reagents and no fecal matter were included throughout the entire extraction process and subsequently sequenced.

Polymerase chain reaction (PCR) amplification and barcoding were conducted in two steps (two-step PCR). In the first step, we targeted the 291 bp fragment of the hypervariable V4 region located in the 16S rRNA gene using the universal bacterial primers 515 F (5′-GTGCCAGCMGCCGCGGTAA-3′) and 806 R (5′-GGACTACHVGGGTWTCTAAT-3′)^81,82^, appended with forward-primer CS1 adapters (CS1-515F) and reverse-primer CS2 adapters (CS2-806R) in order to use Fluidigm chemistry (Access Array System for Illumina Sequencing Systems, Fluidigm Corporation). PCR reactions of 10 µL consisted of 200 nM primers (pooled forward and reverse primers), 5 µL AmpliTaq Gold 360 Master Mix, 1 µL extracted DNA sample, 1 µL DNA template (5–10 ng), and dH_2_0. PCR conditions were as follows: initial denaturation at 95 °C for 10 min, 30 cycles at 95 °C for 30 sec for denaturation, 60 °C for 30 sec for annealing, and 72 °C for 45 sec for elongation, followed by a final elongation at 72 °C for 10 min. PCR success for each individual sample was verified using gel electrophoresis.

In the second PCR step, the CS adapters were attached to sample-specific primer pairs that contained 10 bp barcodes and adapter sequences used for Illumina sequencing. PCR reactions of 20 µL consisted of 4 µL (400 nM) barcode primers (pooled forward and reverse primers), 10 µL AmpliTaq Gold 360 Master Mix, 3 µL amplified DNA from PCR step one, and dH_2_0. PCR conditions were as above, but included 10 cycles instead of 30. Throughout all PCR steps, negative controls containing only the reagents were included and subsequently sequenced. A detailed description of the library preparation is provided in ref. ^83^. In short, 403 fecal samples, 12 extraction blanks, and eight PCR controls were paired-end sequenced using our in-house Illumina MiSeq sequencing platform at the Institute of Evolutionary Ecology and Conservation Genomics, Ulm University, Germany.

### Bioinformatics

To process the reads from Illumina amplicon sequencing, we used the DADA2 plug-in within QIIME 2 (version 2018.6.0)^84^, which encompasses primer removal, denoising, chimera removal, and merging of paired-end reads^85^. DADA2 detects rare variants that differ by only one nucleotide and assembles these into ASVs^86^. The median sequencing depth after DADA2 was just under 26,000 reads per sample. Following denoising, ASVs were taxonomically assigned using the regularly updated SILVA database (v128)^87–89^ via the *classify-sklearn* function in QIIME 2 with its default confidence value settings (0.7)^90^. ASVs which could not be assigned to any known bacterial sequences at the domain and phylum levels, as well as ASVs identified as chloroplast or mitochondrial sequences were excluded from the analysis. An unrooted, phylogenetic tree was then constructed using FastTree (version 2.1.10 Double precision)^91^ and rooted in Dendroscope (version 3.5.10)^92^ using an added archaeon sequence (accession number: KU656649) as the outgroup that was later removed. The sample metadata, the taxonomy table including read counts, and the rooted tree were imported into R^93^ (version 3.6.1) using the phyloseq package^94^ (version 1.28.0). All further analyses, unless otherwise stated, were performed using R^93^.

Once imported from QIIME 2 into R, the extraction blanks and PCR controls contained 90 out of a total of 5633 taxa. Of the 90 taxa, 65 were unique to the blanks/controls and subsequently removed from the dataset. We filtered all ASVs with fewer than 20 reads in total (total abundance) and that were present in less than 2% of all samples (prevalence) from the dataset prior to further downstream analyses. This removed 6 of the remaining 25 ASVs in the blanks/controls. Using prevalence-based contaminant identification with the default threshold of 0.1 from the decontam package^95^ (version 1.4.0), three of the remaining 19 ASVs were identified as possible contaminants and subsequently removed from the dataset. We used both QIIME 2 and the rarecurve function from the vegan package^25^ (version 2.5.5) to generate alpha diversity rarefaction curves, which yielded comparable results. Based on these curves, we set the sequencing depth threshold at 5000 reads per sample and thus eliminated samples with fewer than 5000 reads from downstream analyses, leaving us with a total of 384 fecal samples.

### Statistics and reproducibility

#### Alpha diversity

To determine intraindividual diversity, we calculated three alpha diversity measures: observed number of ASVs (observed ASVs), Shannon diversity^96^, which takes abundance into account, and Faith’s phylogenetic diversity (PD), which also accounts for phylogeny^97^. Using generalized linear mixed models (GLMMs) from the lme4 package^98^ (version 1.1.21), we modeled alpha diversity according to landscape (landscape C, landscape I, and landscape A) and sequencing depth to account for differential sequencing effort between samples^99^. In addition, we included the variables sex (female, male, or unknown) and field season (2013/2014, 2014/2015, or 2016/2017), as there is evidence to suggest that these factors could influence the gut microbiome^100–102^. To facilitate model convergence, sequencing depth was scaled. To control for the lack of independence between study sites and extraction batches (two extraction batches), we set study site nested within landscape as well as extraction batch as (separate) random factor variables. To ensure that estimates were not inflated by collinearity between explanatory variables, we checked variance inflation factors, which were all below a value of two, indicating low collinearity^103^. We used a negative binomial error distribution when modeling the count data (observed ASVs) and a gamma distribution with a log link function when modeling the continuous data (Shannon diversity and Faith’s PD). Model selection was based on the information-theoretic (IT) approach using a second order Akaike’s information criterion corrected for small sample sizes (AIC_C_) as an information criterion and Akaike weights (ω) to determine model support^24^. For all GLMMs, we report both conditional and marginal coefficients of determination of each model (R^2^_GLMM(c)_, which explains the variance of both the fixed and random factors, and R^2^_GLMM(m)_, which explains the variance of the fixed factors only)^104^, which we calculated as the variance explained by the best model, the ΔAIC_C_, conditional parameter estimates (*β*), and 95% confidence intervals (95% CI) using model averaging with a cumulative AIC_C_ω of 95%^24^. In the main body of the article, we present back-transformed parameter estimates and 95% confidence intervals for models with a log link function, while the log transformed versions of these values are presented in the Supplementary Data. Finally, we tested for spatial autocorrelation between the capture sites using a spatial exponential covariance structure on the scaled capture site coordinates in each of the three alpha diversity models using the glmmTMB package^105^ (version 0.2.3).

#### Beta diversity

To assess the gut bacterial community composition between individuals, we calculated weighted and unweighted UniFrac distance matrices using the phyloseq package^94^, which both take phylogenetic relatedness into consideration and, in the case of weighted UniFrac, weighs this information according to abundances^106,107^. We tested for differences in beta diversity between the three landscapes using the permutational multivariate analysis of variance (PERMANOVA) test implemented in the *adonis* function of the vegan package^25,108^. The fixed variables in our full model were: landscape, sex, field season, extraction batch, and sequencing depth. In addition, we nested study site within landscape and passed the factor site through the ‘strata’ argument to block permutations within this nested group. We retained extraction batch in our full model to statistically account for its model support (Supplementary Data 6 and 7). We then passed every possible combination of our full model through the *adonis* function to generate a model selection table sorted according to AIC_C_ values (as described above), which we calculated using the AIC_C_ equation^24^ with input values from the ‘residuals’ and ‘sum of squares’ output of the *adonis* function. We report coefficients of determination of each model (R^2^). We also report Cohen’s *d* effect sizes and 95% confidence intervals^26,27^ calculated using coordinates from the first two PCoA axes^28^. In addition to building a model selection table sorted according to AIC_C_ values, we also subjected this PERMANOVA test to null hypothesis significance testing with 9999 permutations and present *p*-values, F-values, and R^2^. We did this because blocking permutations within capture sites using the ‘strata’ argument in *adonis* only affects *p*-values. This means that in our AIC_C_ calculations we are only partially able to account for any potential spatial effects driven by capture sites by nesting capture site within landscape.

We investigated the homogeneity of the variances of each landscape using the PERMDISP2 test implemented in the *betadisper* function of the vegan package using the distance to the spatial median within study sites^25,29^. Because PERMDISP2 is sensitive to unbalanced study designs, using the distance to centroid within study site enables to accurately reflect the strong effect of study site-specific heterogeneity (while retaining variation within and across sites) and to control for bias in estimates due to variation in sample sizes between study sites^29,109^. To account for bias due to small sample sizes within study sites, we only included study sites for which we had data for 15 or more individuals (C1–C4 *n*_*C*_ = 89, A2–A3 *n*_*A*_ = 107, and I1, I3–I6 *n*_*I*_ = 126, *n*_*total*_ = 322 individuals)^25,109^. We then modeled the distances to the centroids using GLMMs from the lme4 package^98^ with a gamma distribution and log link function for continuous data. The explanatory variables and random factor variables were the same as described above for the alpha diversity metrics, as was the model selection process and the testing for spatial autocorrelation between the capture sites.

Because population density can affect the gut microbiome in its diversity and structure^110^, we initially included this explanatory variable (number of *P. semispinosus* individuals per hectare, scaled) in our alpha and beta diversity analyses. However, controlling for host density did not quantitatively change our results (compare Supplementary Data 1-4 and 6-11 to Supplementary Data 13-22) and, in order to not overparameterize our models, we chose to exclude this variable in our final analysis, though we present the results in the Supplementary Information.

#### Differential abundance analysis

For an in-depth investigation of the ASVs driving the differences between landscapes, we implemented the analysis of composition of microbiomes (ANCOM) test, which was developed specifically for microbiome analyses and which we chose because of its merits of being conservative and boasting low false discovery rates compared to other differential abundance tests^30,111^. Since the results from both the PERMANOVA and PERMDISP2 tests showed that there were no statistically supported differences in beta diversity between the protected continuous forests (landscape C) and the protected forested islands (landscape I; see Results), there was no biological reason to treat these landscapes differently and, thus, we pooled these two landscapes together to compare them against the unprotected forest fragments embedded in an agricultural landscape (landscape A) to understand the effects of additional anthropogenic disturbance. Since landscape was the only fixed parameter to consistently affect beta diversity and to take full advantage of ANCOM’s strength as a nonparametric differential abundance test (i.e., a conservative test that does not make any assumption of parametric distributions)^30^, we ran the function with only landscape as an explanatory variable. As is recommended for large microbiome datasets, we ran ANCOM choosing a moderate correction parameter, which applies the Benjamini–Hochberg (BH) procedure to correct for multiple testing^30,112^. In addition, we determined ASVs for which the null hypothesis was rejected with a rate of at least 95% (cutoff value *w*_*0*_ = 0.95) to be differentially abundant. In order to plot the differentially abundant ASVs in a volcano plot, we adapted code from the QIIME 2 plug-in for compositional data analysis *q2-composition*^84^ to run in R and extracted F values from the ANOVA ran on each ASV based on centered log-ratio transformations (clr) of the ASV table. We also extracted model parameter estimates and standard errors in order to show which ASVs were more or less abundant in each landscape type.

### Predicting microbial genomic functional potential

After establishing which ASVs were differentially abundant between the disturbed and undisturbed landscapes, we implemented the stand-alone PICRUSt2^31–34^ (version 2.1.4-b) to predict metagenomes based on our 16S rRNA gene amplicons. To determine which predicted pathways were differentially abundant between the disturbed and undisturbed landscapes, we first generated unstratified pathway abundances (i.e., a “compressed” table listing the predicted abundances of each pathway, combining all the ASVs contributing to each pathway) based on community-wide pathway abundances (i.e., abundances per pathway versus per ASV) and input these into ANCOM using the same parameters and procedure as described above. To elucidate which ASVs within each of the identified pathways were differentially abundant between the disturbed and undisturbed landscapes, we then generated stratified pathway abundances (i.e., an “expanded” table listing the predicted abundances of each ASV within each pathway) based on community-wide pathway abundances. Due to the large size of our dataset, we were forced to divide our dataset into smaller parts based on samples (not ASVs), running approximately 100 samples in each run. We then combined the outputted tables of each run and filtered the combined table according to the previously identified differentially abundant pathways. The table of predicted abundances for each pathway was then input into ANCOM using the same parameters and procedure as described above. We also added taxonomic assignments to each ASV and removed pathways that are not known to occur in bacteria. We visualized the results as described in the previous paragraph, resulting in one plot per pathway showing, for each pathway, which ASVs were more or less abundant in which landscape along with the taxonomic identification for each ASV.

### Reporting summary

Further information on research design is available in the Nature Research Reporting Summary linked to this article.

## Supplementary information

Supplementary Information

Description of Additional Supplementary Files

Supplementary Data

Reporting Summary

## Data Availability

Microbiome sequences are deposited on NCBI under the accession code PRJNA715350 (https://www.ncbi.nlm.nih.gov/bioproject/PRJNA715350).

## Code availability

The scripts for our analysis are stored on GitHub (https://github.com/gfackelmann/human-encroachment-into-wildlife-gut-microbiomes) and archived in Zenodo under the citable 10.5281/zenodo.4725220^113^.
