# Supplementary material for: Human encroachment into wildlife gut microbiomes
Source: Commun Biol. 2021 Jun 25;4:800. doi: 10.1038/s42003-021-02315-7 (PMC8233340; doi:10.1038/s42003-021-02315-7)
Supplement: Supplementary file 1 — Supplementary Information [file 42003_2021_2315_MOESM1_ESM.pdf]

Human encroachment into wildlife gut microbiomes

Gloria Fackelmann, Mark A. F. Gillingham, Julian Schmid, Alexander Christoph Heni, Kerstin Wilhelm, Nina Schwensow, Simone Sommer

Supplementary Information

Supplementary Figures 1-10

Supplementary Results

Supplementary References

Supplementary Figure 1: Map of the study area encompassing locations of the 17 study sites in Panama, Central America.

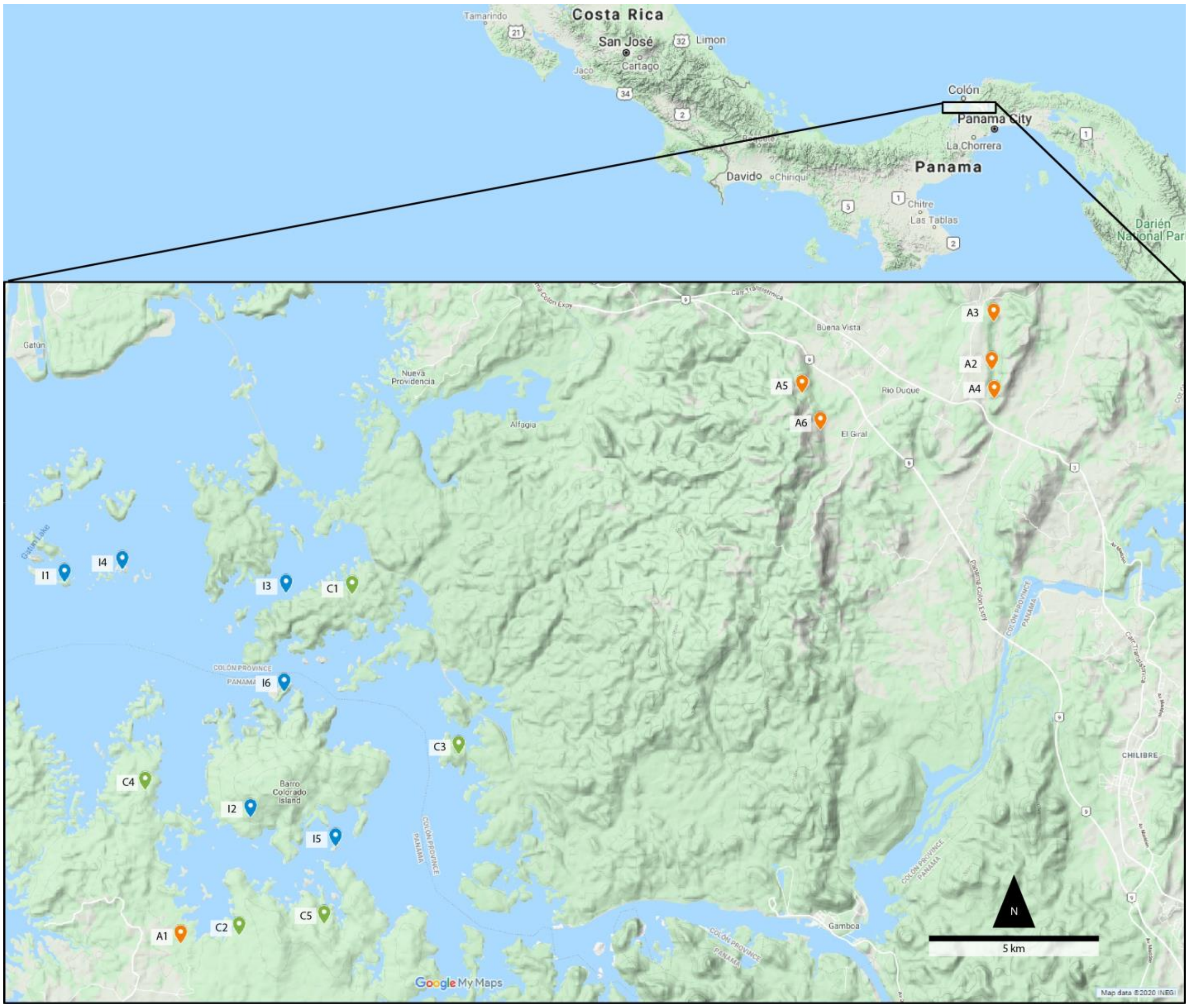

Proechimys semispinosus individuals were trapped in protected continuous tropical forests with no human contact (control study sites C1-C5 in green), in protected fragmented tropical forests on islands with no human contact that allow the study of the effects of fragmentation alone (study sites I1-I6 in blue), and in unprotected, tropical forest fragments embedded in an agricultural matrix that are subjected to additional anthropogenic disturbance i.e. contact with humans, domesticated animals, invasive species and pathogens (study sites A1-A6 in orange).

Supplementary Figure 2: Schematic representation of the three landscapes (C, I, and A) defined in this study.

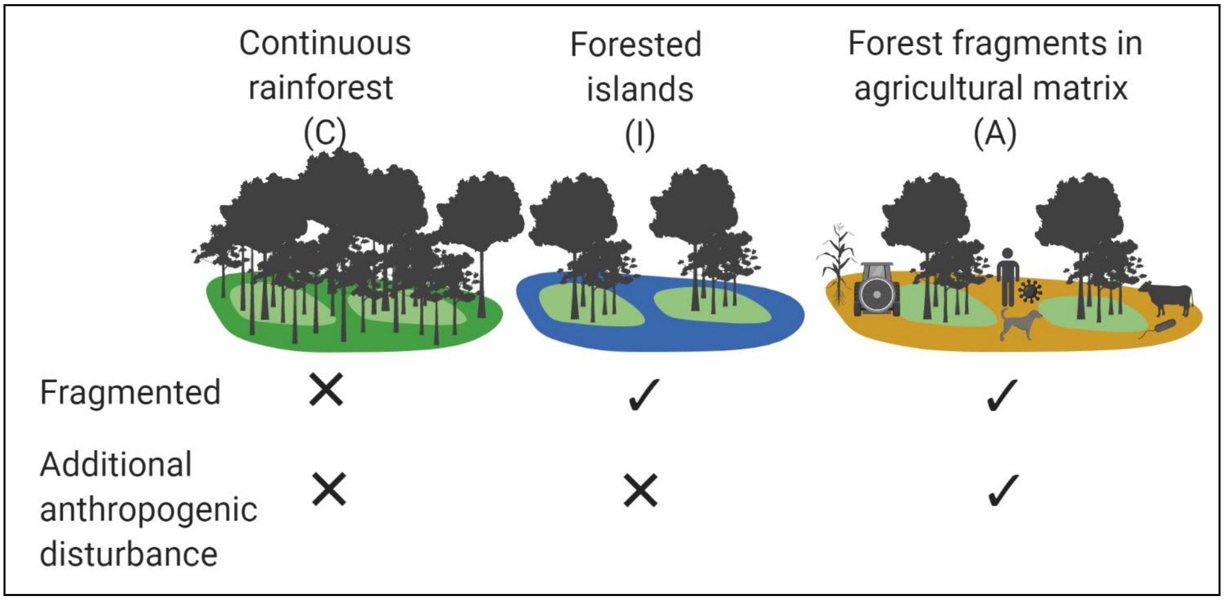

The forest patches across all three landscapes are lowland tropical rainforests, but the landscapes differ in the matrix in which these patches are embedded (forest, water, or agriculture). The continuous forest (C) landscape is characterized by its protected continuous rainforest (i.e. rainforest "patches" embedded in a matrix of rainforest), whilst the forested islands (I) are protected forest patches embedded in a matrix of water (the islands are located in the Panama Canal), and the forest fragments (A) are unprotected forest patches embedded in a matrix of agriculture. Consequently, landscape C is neither fragmented nor is it subjected to anthropogenic disturbance because its forests are protected, whilst landscape I is fragmented but not subjected to any additional anthropogenic disturbance because its forests are also protected. Landscape A, however, is both fragmented and subjected to additional anthropogenic disturbance in the form of contact with humans, domesticated animals, invasive species, and their pathogens. Created with BioRender.com.

Supplementary Figure 3: Landscape influences gut microbial alpha diversity in the generalist spiny rat *P. semispinosus*.

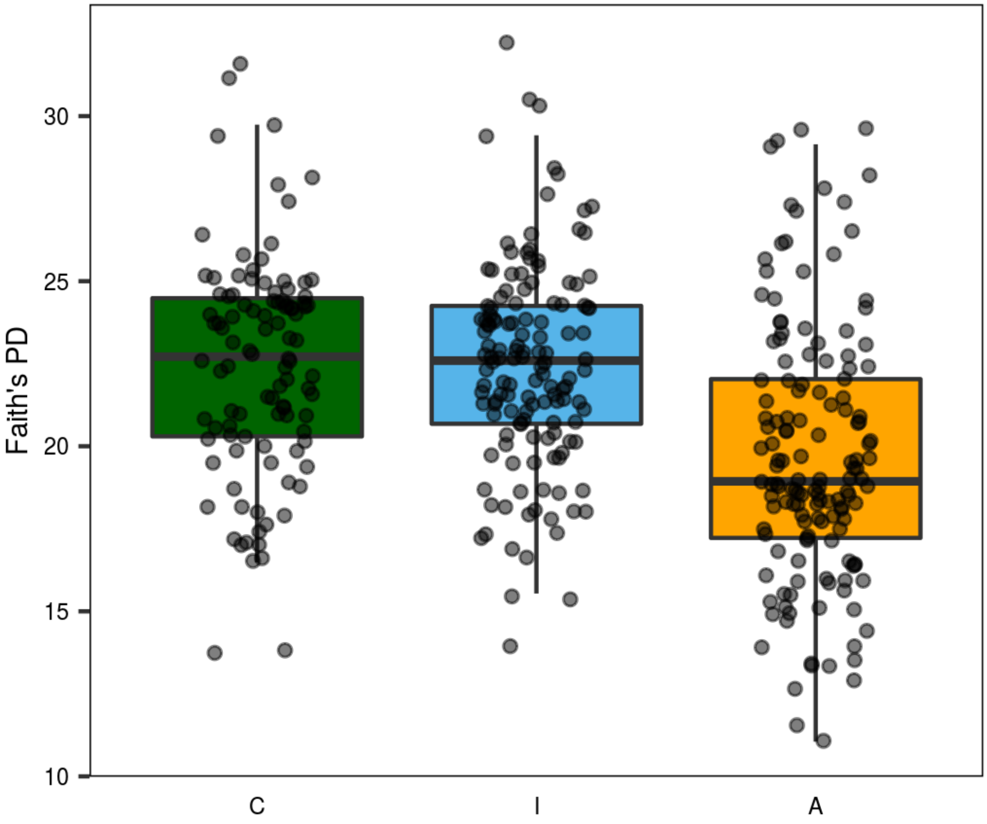

Boxplots (center line, median; hinges, 25th and 75th percentiles; whiskers, 1.5 x IQR) of the gut microbial alpha diversity measured using Faith's phylogenetic diversity (PD) within spiny rat individuals (*n* = 384 individuals) inhabiting landscape C (green, *n* = 103 individuals), landscape I (blue, *n* = 136 individuals), and landscape A (orange, *n* = 145 individuals).

Supplementary Figure 4: Landscape influence on inter-individual gut microbial diversity in the generalist spiny rat *P. semispinosus* apparent across replicate study sites.

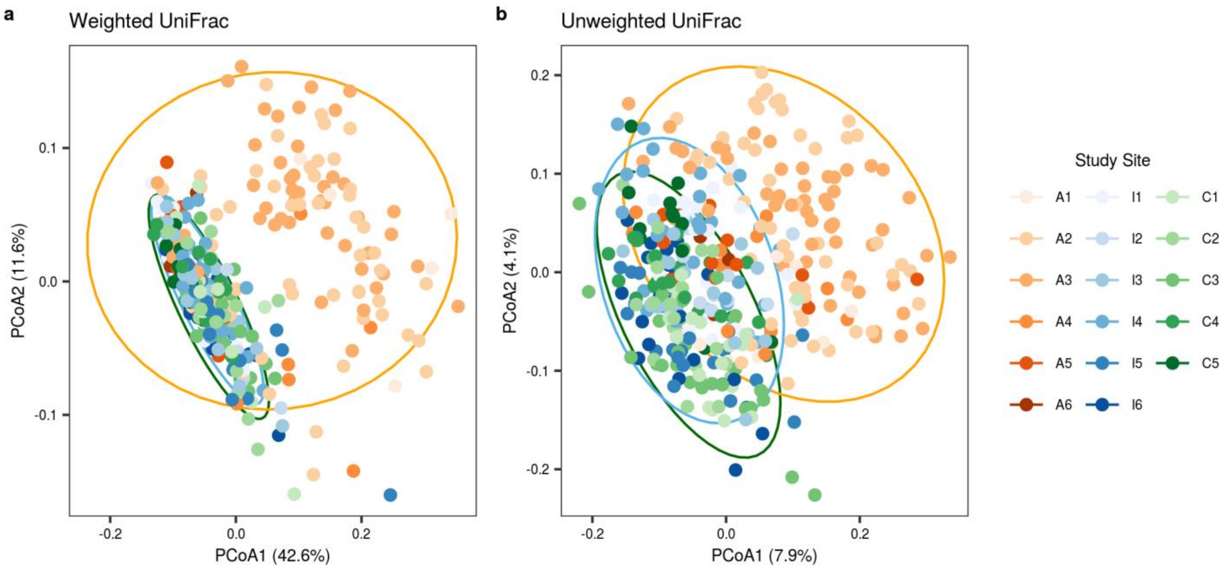

a,**b** Principal coordinates analysis (PCoA) plots of the diversity between spiny rat individuals (*n* = 384 individuals) accounting for phylogenetic diversity and weighted (**a**) and unweighted (**b**) for abundance using UniFrac distances. Each dot represents a *P. semispinosus* individual sampled in landscape C (shades of green, *n* = 103 individuals), landscape I (shades of blue, *n* = 136 individuals), or landscape A (shades of orange, *n* = 145 individuals) and colored according to study site. Ellipses indicate 95% confidence intervals.

Supplementary Figure 5: Gut microbial composition of spiny rats *P. semispinosus* in each of the three landscapes (C, I, and A).

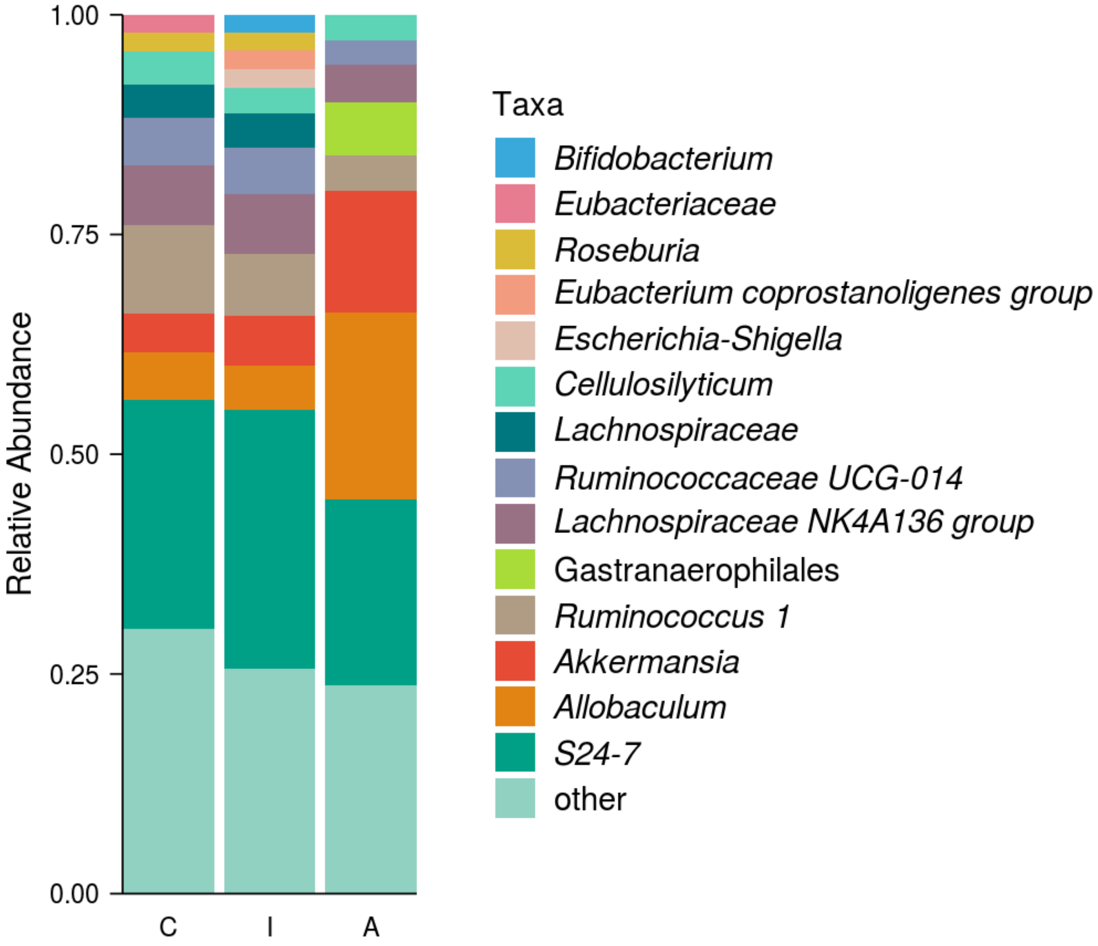

Shown are the relative abundances of taxa whose relative abundances were above 2% (those with a lower relative abundance were grouped together as "other") grouped together according to landscape (landscape C, *n* = 103 individuals; landscape I, *n* = 136 individuals; and landscape A, *n* = 145 individuals). Each taxon represents the closest taxonomic rank to genus as was classifiable.

Supplementary Figure 6: ASVs associated with the predicted pathway 1861 (formaldehyde assimilation II – RuMP Cycle) that were differentially abundant between protected landscapes (C and I) and anthropogenically more disturbed landscape A.

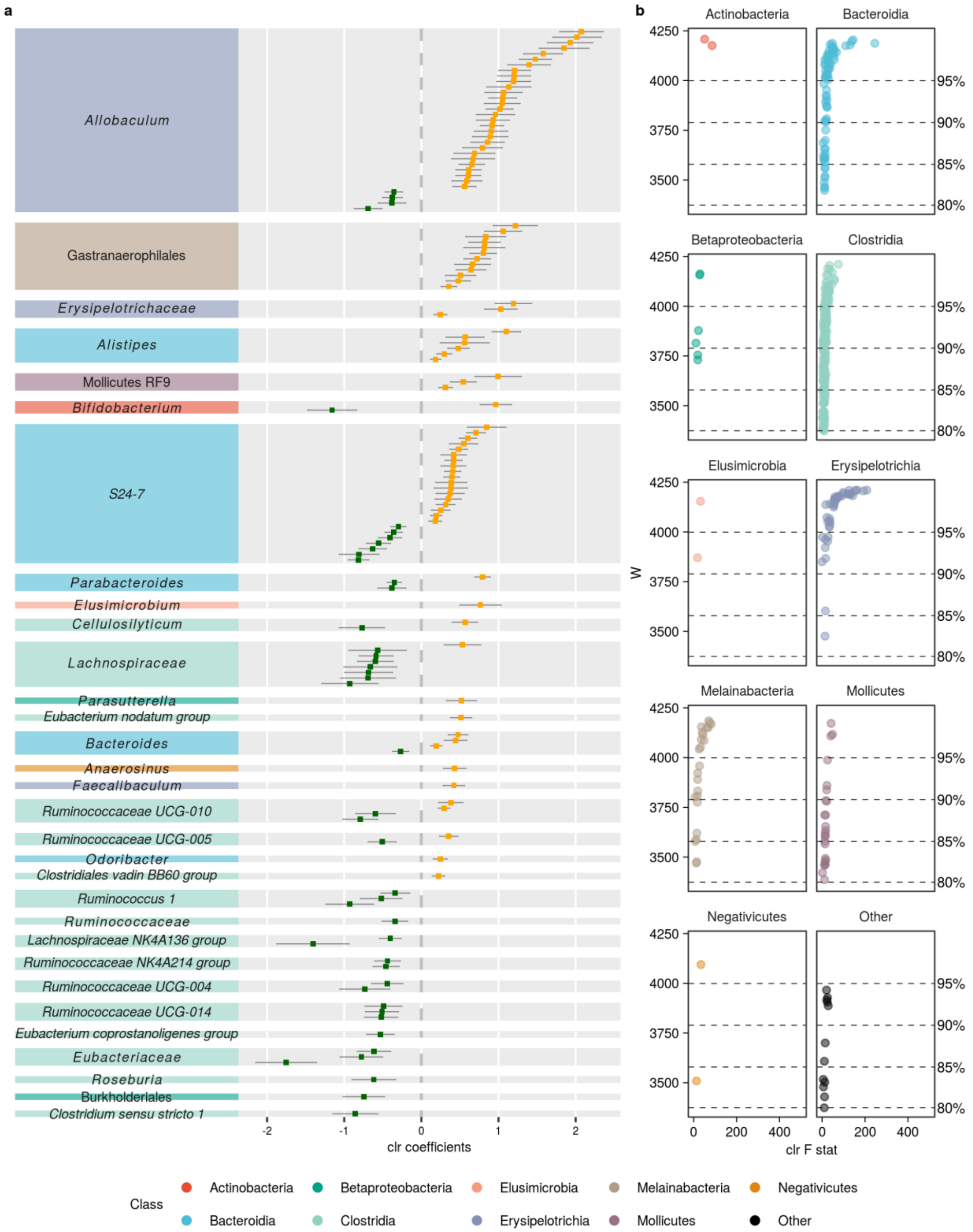

a The 134 ASVs out of a total of 4,213 ASVs associated with the predicted pathway 1861 that were determined to be differentially abundant (at $w > 95\%$) between landscapes C and I (green) and landscape A (orange). Each dot is an ASV plotted in decreasing order by its coefficient with confidence intervals as 1.96 times the standard error. Dots to the right of center zero in orange represent ASVs over-represented in landscape A, whilst dots to the left in green represent ASVs over-represented in landscapes C and I. ASVs are grouped by most detailed taxonomic assignment. Taxa are colored according to class. **b** Volcano plots of differentially abundant ASVs (at $w > 80\%$) and those leading up to the determined cutoff value $w_0 = 0.95$ are displayed in **a**. Each dot represents an ASV plotted by its F statistic as a measure of effect size and by its W value, which is a count of how many times the null hypothesis was rejected for that particular ASV. The dots are colored by taxonomic class as in **a**.

Supplementary Figure 7: ASVs associated with the predicted pathway 2941 (L-lysine biosynthesis II) that were differentially abundant between protected landscapes (C and I) and anthropogenically more disturbed landscape A.

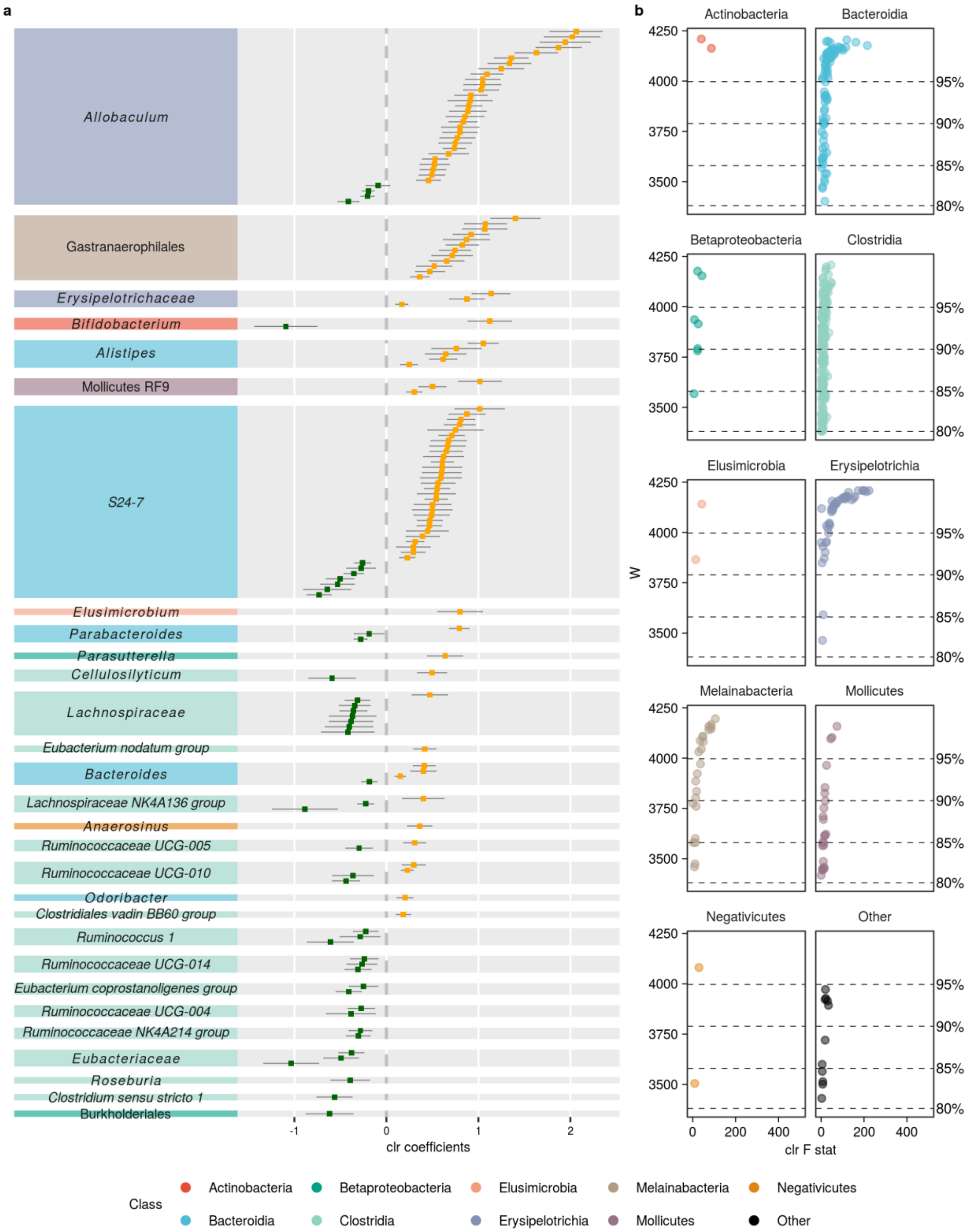

a The 134 ASVs out of a total of 4,213 ASVs associated with the predicted pathway 1861 that were determined to be differentially abundant (at $w > 95\%$) between landscapes C and I (green) and landscape A (orange). Each dot is an ASV plotted in decreasing order by its coefficient with confidence intervals as 1.96 times the standard error. Dots to the right of center zero in orange represent ASVs over-represented in landscape A, whilst dots to the left in green represent ASVs over-represented in landscapes C and I. ASVs are grouped by most detailed taxonomic assignment. Taxa are colored according to class. **b** Volcano plots of differentially abundant ASVs (at $w > 80\%$) and those leading up to the determined cutoff value $w_0 = 0.95$ are displayed in **a**. Each dot represents an ASV plotted by its F statistic as a measure of effect size and by its W value, which is a count of how many times the null hypothesis was rejected for that particular ASV. The dots are colored by taxonomic class as in **a**.

Supplementary Figure 8: ASVs associated with the predicted teichoic acid pathway (teichoic acid (poly-glycerol) biosynthesis) that were differentially abundant between protected landscapes (C and I) and anthropogenically more disturbed landscape A.

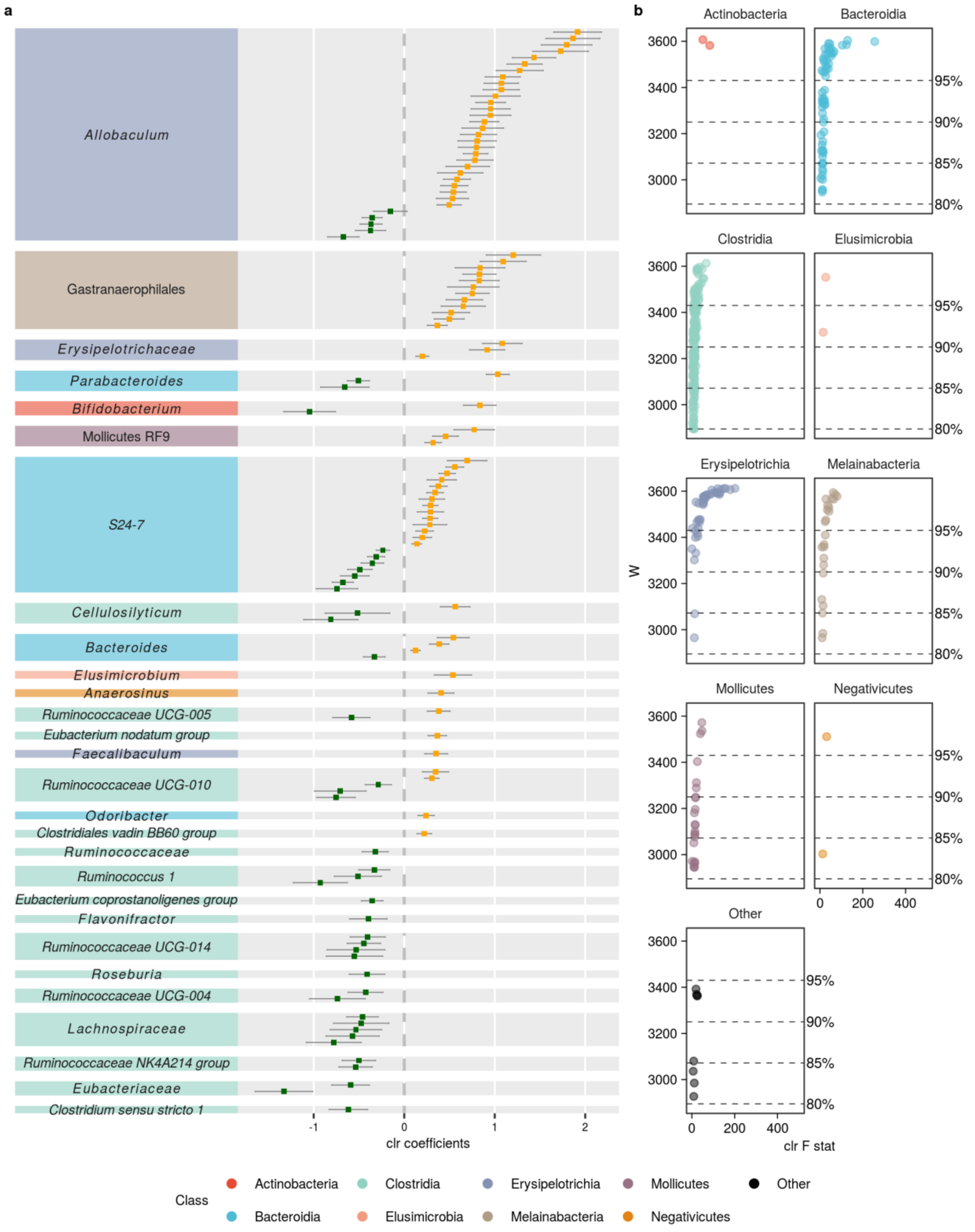

a The 134 ASVs out of a total of 4,213 ASVs associated with the predicted pathway 1861 that were determined to be differentially abundant (at *w* > 95%) between landscapes C and I (green) and landscape A (orange). Each dot is an ASV plotted in decreasing order by its coefficient with confidence intervals as 1.96 times the standard error. Dots to the right of center zero in orange represent ASVs over-represented in landscape A, whilst dots to the left in green represent ASVs over-represented in landscapes C and I. ASVs are grouped by most detailed taxonomic assignment. Taxa are colored according to class. **b** Volcano plots of differentially abundant ASVs (at *w*> 80%) and those leading up to the determined cutoff value w_0 = 0.95 are displayed in **a**. Each dot represents an ASV plotted by its F statistic as a measure of effect size and by its W value, which is a count of how many times the null hypothesis was rejected for that particular ASV. The dots are colored by taxonomic class as in **a**.

Supplementary Figure 9: ASVs associated with the predicted pathway 7210 (pyrimidine deoxyribonucleotides biosynthesis from CTP) that were differentially abundant between protected landscapes (C and I) and anthropogenically more disturbed landscape A.

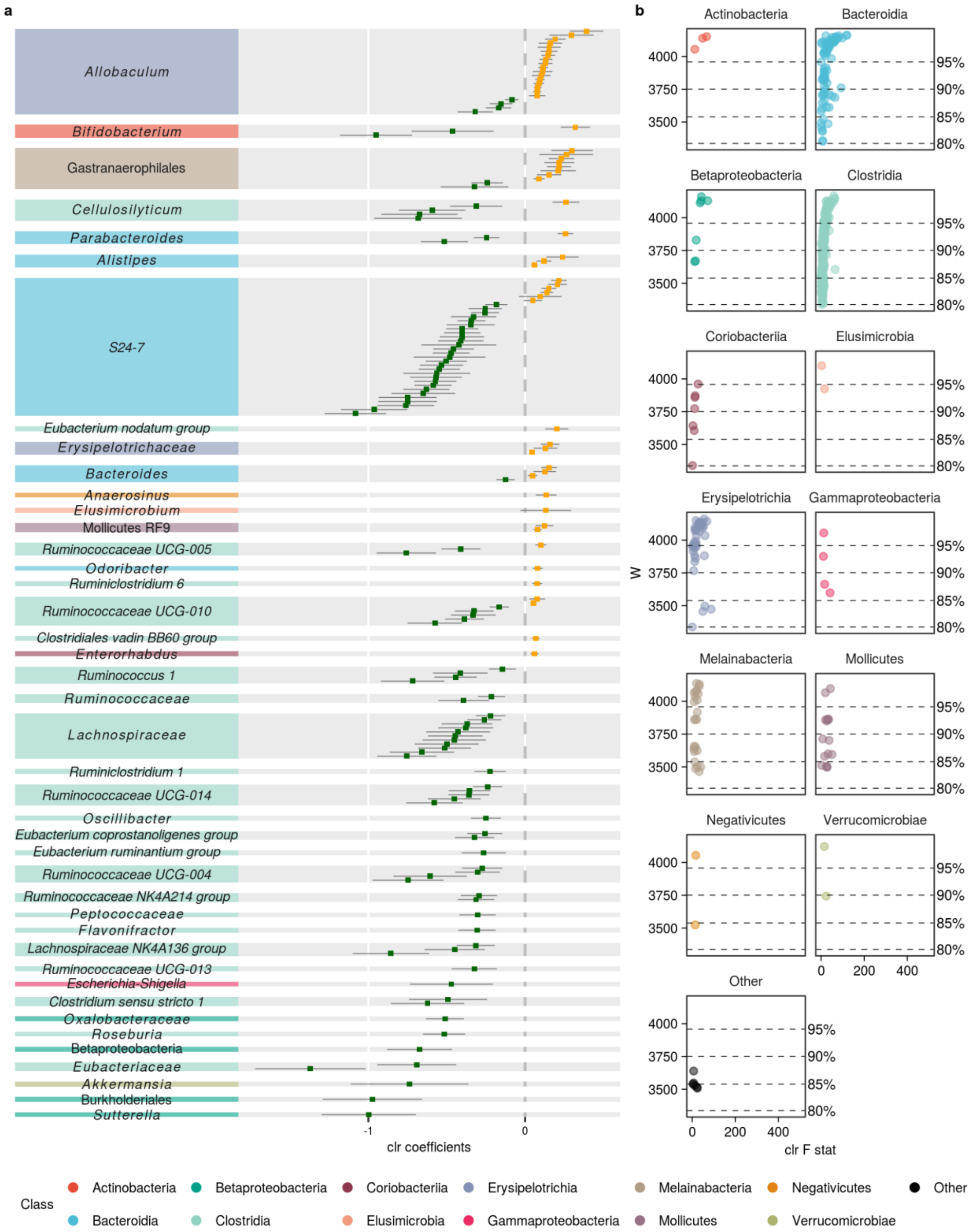

a The 134 ASVs out of a total of 4,213 ASVs associated with the predicted pathway 1861 that were determined to be differentially abundant (at w > 95%) between landscapes C and I (green) and landscape A (orange). Each dot is an ASV plotted in decreasing order by its coefficient with confidence intervals as 1.96 times the standard error. Dots to the right of center zero in orange represent ASVs over-represented in landscape A, whilst dots to the left in green represent ASVs over-represented in landscapes C and I. ASVs are grouped by most detailed taxonomic assignment. Taxa are colored according to class. **b** Volcano plots of differentially abundant ASVs (at w> 80%) and those leading up to the determined cutoff value w_0 = 0.95 are displayed in **a**. Each dot represents an ASV plotted by its F statistic as a measure of effect size and by its W value, which is a count of how many times the null hypothesis was rejected for that particular ASV. The dots are colored by taxonomic class as in **a**.

Supplementary Figure 10: Gut microbial alpha diversity as measured by the observed number of ASVs increases with increasing host density in the generalist spiny rat *P. semispinosus* in each of the three landscapes (C, I, and A).

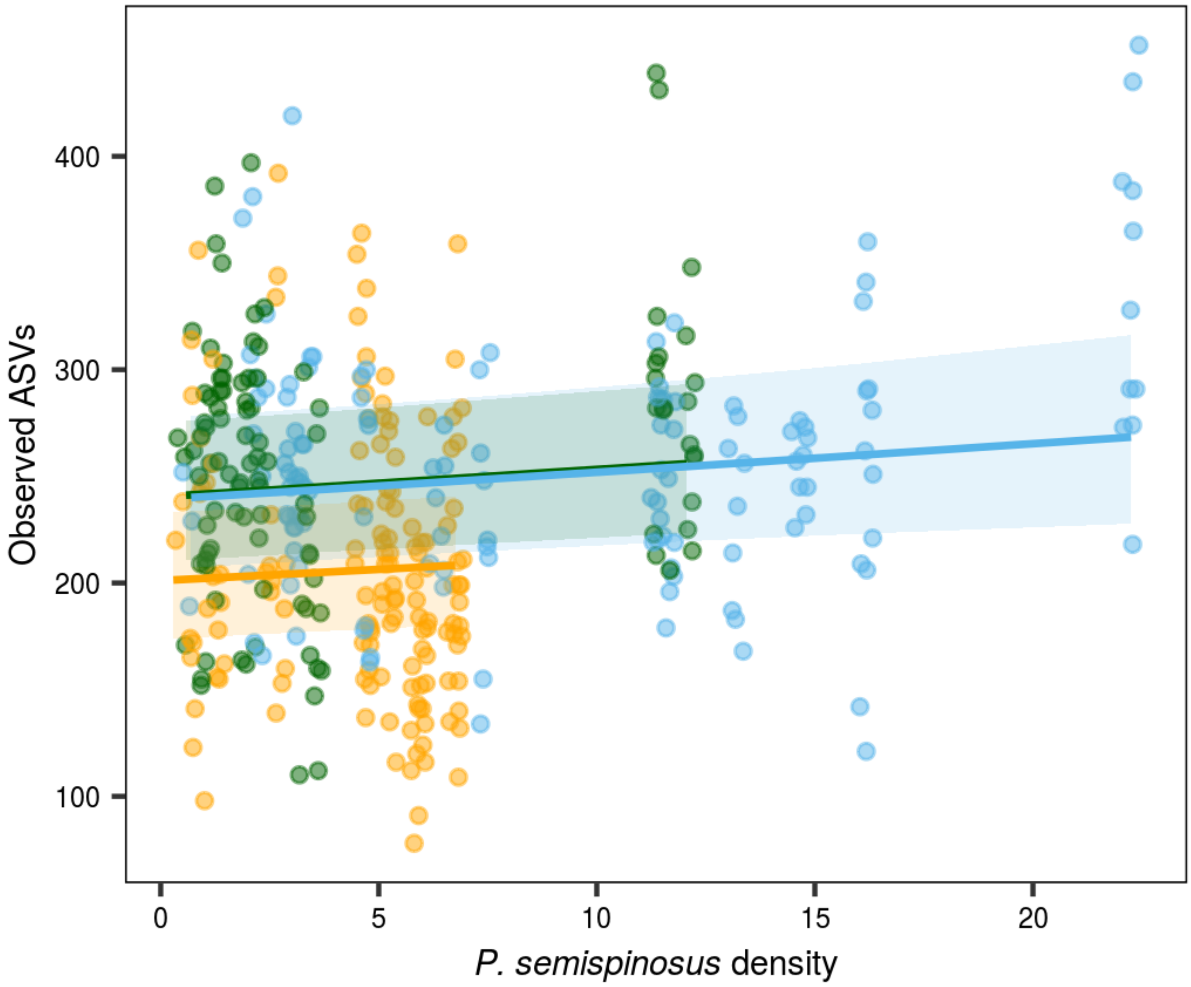

Plotted is the number of observed ASVs per spiny rat individual (*n* = 384 individuals) according to the density of spiny rats (individuals per hectare) in the respective capture sites and colored according to landscape (landscape C, green, *n* = 103 individuals; landscape I, blue, *n* = 136 individuals; and landscape A, orange, *n* = 145 individuals). The dark lines denote the predicted values based on the top ranked model according to IT model selection. The shaded areas around the dark lines represent the upper and lower 95% confidence intervals.

Supplementary Results

Effect of host population density on the gut microbiome

Considering the coprophagic nature of rodents[1], a higher host density could increase the fecal-oral transfer of microbes, thus leading to higher levels of alpha diversity in individuals inhabiting high-density landscapes[2]. We examined this by using generalized linear mixed models in conjunction with IT model selection (see Methods). We found support for an effect of host density on the observed number of ASVs ($\Delta AIC_C = 2.41$, Supplementary Data 13), which showed a weak positive relationship between host density and alpha diversity ($\beta_{density} = 0.025$ (95% CI = 0.001 to 0.048); $R^2_{GLMM(m)density} = 0.002$, $R^2_{GLMM(c)density} = 0.001$; Supplementary Data 13 and 16; Supplementary Figure 10), but little support for this effect on Shannon diversity ($\Delta AIC_C = 0.00$, $\beta_{density} = 4.517$ (95% CI = 4.250 to 4.800), Supplementary Data 14 and 16) and on Faith's phylogenetic diversity ($\Delta AIC_C = 0.97$, $\beta_{density} = 22.22$ (95% CI = 19.16 to 25.78), Supplementary Data 15 and 16). Next, we investigated if host density impacts beta diversity in *P. semispinosus* by (1) testing for shifts in gut community composition using the permutational multivariate analysis of variance (PERMANOVA) test and (2) testing for differences in homogeneity of variance between the landscapes using the distances calculated by PERMDISP2[3]. Using both weighted and unweighted UniFrac distances, there was no support for an effect of host density on gut microbial beta diversity using the IT approach (Supplementary Data 17-18 and 20-22). In addition to building a model selection table sorted according to AIC_C values, we also subjected this PERMANOVA test to null hypothesis significance testing with 9,999 permutations because blocking permutations within capture sites using the 'strata' argument in *adonis* only affects *p*-values, meaning we are only partially able to account for any potential spatial effects driven by capture sites in our AIC_C calculations by nesting capture site within landscape (see Methods). Using null hypothesis significance testing, host density did have a significant effect on beta diversity community composition (weighted UniFrac: $p = 0.048$ for 9,999 permutations, $R^2 = 0.006$, $F = 2.93$;

unweighted UniFrac: $p = 0.015$ for 9,999 permutations, $R^2 = 0.007$, F = 3.29; Supplementary Data 19).

Though our study provides limited support for an effect of host density on beta diversity, it does support this effect on alpha diversity. In all three landscapes, greater host density was associated with increased intra-individual diversity, however, only when accounting for species richness and not abundance. Sharing of rare taxa could have a greater impact on species richness than on abundance, as opposed to the transmission of taxa already in greater abundances across the population. Thus, the increase in alpha diversity is likely driven by the sharing of rare taxa as opposed to highly abundant ones. Increased population density, especially in coprophagic animals, can be seen as a proxy for frequency of contact between individuals[4] and thus microbial sharing between individuals[2]. Consequently, our results show that host density on the landscape level can shape the gut microbial diversity within individuals.

Differential abundance analysis

The 30 ASVs that were over-represented solely in the anthropogenically disturbed landscape A ranged across eight different classes and belonged to the order Gastranaerophilales (12 ASVs), the genus *Alistipes* (5 ASVs), the family *Erysipelotrichaceae* (3 ASVs), the order Mollicutes RF9 (3 ASVs), and the following taxa with each one ASV: *Clostridiales* vadin BB60 group family, genera *Elusimicrobium*, *Parasutterella*, *Anaerosinus*, *Faecalibaculum*, *Odoribacter*, and species *Eubacterium nodatum* group. In contrast, the 19 ASVs that were over-represented solely in the protected landscapes C and I ranged across only two classes, namely Betaproteobacteria and Clostridia. Within the Betaproteobacteria, these ASVs belonged to the order Burkholderiales (1 ASV) and, within that order, to the *Oxalobacteraceae* family (1 ASV). Within the Clostridia class, the family with the greatest number of ASVs over-represented in the study sites of landscapes C and I was *Ruminococcaceae* (1 ASV) with the genera *Ruminococcaceae UCG-014* (4 ASVs), *Ruminococcaceae NK4A214* group (2 ASVs), and *Ruminococcaceae* UCG-004 (1 ASV), followed

by the family *Eubacteriaceae* (2 ASVs) with the genus *Eubacterium coprostanoligenes* group (1 ASV), and the genera *Roseburia* (2 ASVs), *Lachnospiraceae NK4A136* group (2 ASVs), *Flavonifractor* (1 ASV), and *Clostridium sensustrincto 1* (1 ASV).

Predicted metagenome functions

Pathway 7210 was the only pathway predominantly over-represented in the protected landscapes (C and I). In addition to this, it was distinguishable from the other three pathways in that the differentially abundant ASVs associated with this pathway belong to three additional classes, which were not common to the other three pathways: Coriobacteriia, Gammaproteobacteria, and Verrucomicrobiae (Supplementary Figure 9). More specifically, the differentially abundant taxa unique to pathway 7210 were: *Ruminiclostridium 6*, *Enterorhabdus*, *Ruminiclostridium 1*, *Oscillibacter*, *Eubacterium ruminantium* group, *Peptococcaceae*, *Ruminococcaceae UCG-013*, *Escherichia-Shigella*, *Oxalobacteraceae*, *Akkermansia*, and *Sutterella*.

In general, the patterns observed based on differentially abundant ASVs are also observed based on predicted pathways: The differentially abundant ASVs belonging to *Odoribacter*, Gastranaerophilales, and members of the Mollicutes RF9 were over-represented in landscape A (Figure 2a) and, similarly, each of the four pathways associated with these taxa were also over-represented in landscape A, almost exclusively with the exception of pathway 7210 (compare Supplementary Figures 6a-8a with Supplementary Figure 9a). In addition, ASVs belonging to *Clostridium sensu strincto 1*, *Roseburia*, and the *Eubacterium coprostanoligenes* group were over-represented in the landscapes C and I (Figure 2a) and, similarly, each of the four predicted pathways associated with these three taxa are also more abundant in landscapes C and I (Supplementary Figures 6a-9a). Moreover, the greatest number of differentially abundant ASVs associated with all four differentially abundant predicted pathways belonged to *Allobaculum* and the *S24-7* family, which again contained some pathway-associated ASVs that were more abundant in the landscapes C and I, whilst other pathway-associated ASVs were more abundant in landscape

A (Supplementary Figures 6a-9a), thus reflecting a very similar pattern as observed based on the differential abundance analysis.

Concerning the functions of each of the four pathways, pathway 7210 represents a salvaging pathway of free bases and nucleosides to biosynthesize pyrimidine nucleotides, which can be preferable to *de novo* biosynthesis pathways that require more energy[5,6]. The teichoic acid pathway encompasses the biosynthesis of teichoic acids[6,7], a group of glycopolymers found either anchored to cell membranes or bound to peptidoglycan cell walls of gram-positive bacteria[8]. As such, they play a key role in pathogenesis and exhibit structural diversity by using a range of carbohydrates, commonly glucose[8]. It has been shown that elevated teichoic acid levels in cell walls of pathogenic bacteria increased their pathogenicity[9] and inhibitors of this pathway are being targeted as potential antibiotics[10]. Pathway 2941 encompasses the acetylase variant of L-lysine biosynthesis belonging to the diaminopimelate (DAP) group i.e. the biosynthesis of the amino acid lysine and its direct precursor meso-diaminopimelate (meso-DAP)[6,11], both of which are key components of bacterial peptidoglycan cell walls in gram-positive and gram-negative bacteria, respectively[12]. As with teichoic acid biosynthesis, the inhibition of lysine biosynthesis in bacteria is also a target for potential antibiotic agents[12]. Pathway 1861 describes the assimilation of the cytotoxic formaldehyde, which can be generated during the oxidation of methane and methanol[6,13] and produced as an end product during the generation of free radicals during inflammation[14]. It has been proposed that bacterial pathogens in particular show a wide range of adaptive responses in dealing with formaldehyde, including horizontal gene transfer aiding in formaldehyde detoxification between bacterial pathogens that are otherwise not known to be methylotrophic.

Supplementary references

1. Soave, O. & Brand, C. D. Coprophagy in animals: a review. *Cornell Vet.* **81**, 357—364

(1991).

2. Li, H. *et al.* Pika population density is associated with the composition and diversity of gut microbiota. *Front. Microbiol.* **7**, 1–9 (2016).

3. Anderson, M. J. Distance-based tests for homogeneity of multivariate dispersions. *Biometrics* **62**, 245–253 (2006).

4. Davis, S., Abbasi, B., Shah, S., Telfer, S. & Begon, M. Spatial analyses of wildlife contact networks. *J. R. Soc. Interface* **12**, (2015).

5. MetaCyc. MetaCyc Pathway: pyrimidine deoxyribonucleotides biosynthesis from CTP. https://biocyc.org/META/NEW-IMAGE?type=PATHWAY&object=PWY-7210&show-citations=T.

6. Caspi, R. *et al.* The MetaCyc database of metabolic pathways and enzymes. *Nucleic Acids Res.* **46**, D633–D639 (2018).

7. MetaCyc. MetaCyc Pathway: poly(glycerol phosphate) wall teichoic acid biosynthesis. https://biocyc.org/META/NEW-IMAGE?type=PATHWAY&object=TEICHOICACID-PWY.

8. Brown, S., Santa Maria, J. P. & Walker, S. Wall teichoic acids of gram-positive bacteria. *Annu. Rev. Microbiol.* **67**, 313–336 (2013).

9. Wanner, S. *et al.* Wall teichoic acids mediate increased virulence in *Staphylococcus aureus*. *Nat. Microbiol.* **2**, 1–12 (2017).

10. Pasquina, L. W., Santa Maria, J. P. & Walker, S. Teichoic acid biosynthesis as an antibiotic target. *Curr. Opin. Microbiol.* **16**, 531–537 (2013).

11. MetaCyc. MetaCyc Pathway: L-lysine biosynthesis II. https://metacyc.org/META/NEW-IMAGE?type=PATHWAY&object=PWY-2941.

12. Hutton, C. A., Perugini, M. A. & Gerrard, J. A. Inhibition of lysine biosynthesis: An evolving antibiotic strategy. *Mol. Biosyst.* **3**, 458–465 (2007).

13. MetaCyc. MetaCyc Pathway: formaldehyde assimilation II (assimilatory RuMP Cycle). https://biocyc.org/META/NEW-IMAGE?type=PATHWAY&object=PWY-1861.

14. Chen, N. H., Djoko, K. Y., Veyrier, F. J. & McEwan, A. G. Formaldehyde stress responses in bacterial pathogens. *Front. Microbiol.* **7**, 1–17 (2016).
