# Supplementary material for: Human encroachment into wildlife gut microbiomes
Source: Commun Biol. 2021 Jun 25;4:800. doi: 10.1038/s42003-021-02315-7 (PMC8233340; doi:10.1038/s42003-021-02315-7)
Supplement: Supplementary file 2 — Description of Additional Supplementary Files [file 42003_2021_2315_MOESM2_ESM.pdf]

Description of Additional Supplementary Files

File Name: Supplementary Data

Descriptions:

Supplementary Data 1: Model selection table of generalized linear mixed models with a negative binomial distribution and observed number of ASVs as a response variable. In the global model, fixed variables are landscape, sequencing depth (scaled), field season, and sex, and random intercepts set for study site nested within landscape and for extraction batch. Models are ranked according to their Akaike information criterion adjusted for small sample sizes (AIC_C) from highest model support (lowest AIC_C value) to lowest model support (highest AIC_C value). The plus sign (+) indicates which variables are included in that specific candidate model. For each candidate model, the degrees of freedom (df), marginal and conditional coefficients of determination ($R^2_{GLMM(m)}$and $R^2_{GLMM(c)}$), log likelihood (logLik), difference in AIC_C to the AIC_C of the top ranked model (ΔAIC_C), and the Akaike weights ($AIC_C\omega$) are shown.

Supplementary Data 2: Model selection table of generalized linear mixed models with a gamma distribution and log link function and Shannon diversity as a response variable. In the global model, fixed variables are landscape, sequencing depth (scaled), field season, and sex, and random intercepts set for study site nested within landscape and for extraction batch. Models are ranked according to their Akaike information criterion adjusted for small sample sizes (AIC_C) from highest model support (lowest AIC_C value) to lowest model support (highest AIC_C value). The plus sign (+) indicates which variables are included in that specific candidate model. For each candidate model, the degrees of freedom (df), marginal and conditional coefficients of determination ($R^2_{GLMM(m)}$ and $R^2_{GLMM(c)}$), log likelihood (logLik), difference in AIC_C to the AIC_C of the top ranked model (ΔAIC_C), and the Akaike weights ($AIC_C\omega$) are shown.

Supplementary Data 3: Model selection table of generalized linear mixed models with a gamma distribution and log link function and Faith's phylogenetic diversity as a response variable. In the global model, fixed variables are landscape, sequencing depth (scaled), field season, and sex, and random intercepts set for study site nested within landscape and for extraction batch. Models are ranked according to their Akaike information criterion adjusted for small sample sizes (AIC_C) from highest model support (lowest AIC_C value) to lowest model support (highest AIC_C value). The plus sign (+) indicates which variables are included in that specific candidate model. For each candidate model, the degrees of freedom (df), marginal and conditional coefficients of determination ($R^2_{GLMM(m)}$ and $R^2_{GLMM(c)}$), log likelihood (logLik), difference in AIC_C to the AIC_C of the top ranked model (ΔAIC_C), and the Akaike weights ($AIC_C\omega$) are shown.

Supplementary Data 4: Alpha diversity generalized linear mixed models' model-averaged conditional parameter estimates and 95% confidence intervals. For each of the three alpha diversity metrics (observed number of ASVs, Shannon diversity and Faith's phylogenetic diversity), generalized linear mixed models were averaged with a cumulative AIC_C weight ($AIC_C\omega$) of 0.95. Each model contained the fixed variables landscape, sequencing depth (scaled), field season, and sex, and random intercepts set for study site nested within landscape and for extraction batch. The explanatory variables listed are those that were retained in the averaged models. For each explanatory variable in each model, we report the parameter estimate (β) and upper and lower 95% confidence intervals (upper/lower 95% CI). Please note that because the models used here were built using a log link function, the parameter estimates and 95% confidence intervals are log transformed.

Supplementary Data 5: Model comparison of alpha diversity models with and without a spatial autocorrelation parameter. The response variables and distributions of these generalized linear mixed models are: the observed number of ASVs in models with a negative binomial distribution, Shannon diversity and Faith's phylogenetic diversity with gamma distribution and log link function. In each model, fixed variables are landscape, sequencing depth (scaled), field season, and sex, and random intercepts set for study site nested within landscape and for extraction batch. For each alpha diversity metric, there is a model with and without an expontential spatial autocorrelation parameter. Within each alpha diversity metric, models are ranked according to their Akaike information criterion adjusted for small sample sizes (AIC_C) from highest model support (lowest AIC_C value) to lowest model support (highest AIC_C value). The plus sign (+) indicates which models included the spatial autocorrelation parameter. For each model, the degrees of freedom (df), log likelihood (logLik), difference in AIC_C to the AIC_C of the top ranked model within an alpha diversity metric (ΔAIC_C), and the Akaike weights ($AIC_C\omega$) are shown.

Supplementary Data 6: Model selection table of generalized linear models with weighted UniFrac distances as a response variable to study shifts in gut community composition using the output from the *adonis* function. In the global model, fixed variables are landscape, study site nested within landscape, sequencing depth, field season, sex, and extraction batch. The nested variable study site is conformably passed through the *strata* argument. Models are ranked according to their Akaike information criterion adjusted for small sample sizes (AIC_C) from highest model support (lowest AIC_C value) to lowest model support (highest AIC_C value). The plus sign (+) indicates which variables are included in that specific candidate model. For each candidate model, the coefficient of determination (R^2), log likelihood (logLik), difference in AIC_C to the AIC_C of the top ranked model (ΔAIC_C), and the Akaike weights ($AIC_C\omega$) are shown.

Supplementary Data 7: Model selection table of generalized linear models with unweighted UniFrac distances as a response variable to study shifts in gut community composition using the output from the *adonis* function. In the global model, fixed variables are landscape, study site nested within landscape, sequencing depth, field season, sex, and extraction batch. The nested variable study site is conformably passed through the *strata* argument. Models are ranked according to their Akaike information criterion adjusted for small sample sizes (AIC_C) from highest model support (lowest AIC_C value) to lowest model support (highest AIC_C value). The plus sign (+) indicates which variables are included in that specific candidate model. For each candidate model, the coefficient of determination (R^2), log likelihood (logLik), difference in AIC_C to the AIC_C of the top ranked model (ΔAIC_C), and the Akaike weights ($AIC_C\omega$) are shown.

Supplementary Data 8: Results from null hypothesis significance testing with weighted and unweighted UniFrac distances as response variables to study shifts in gut community composition using the *adonis* function. In the model, explanatory variables are (in order): study site nested within landscape, landscape, field season, sex, sequencing depth, and extraction batch. The nested variable study site is conformably passed through the *strata* argument. For each explanatory variable, the degrees of freedom (df), F-values, coefficients of determination (R^2), and *p*-values for 9,999 permutations are shown.

Supplementary Data 9: Model selection table of generalized linear mixed models and weighted UniFrac distances to the study site centroids as a response variable to study differences in dispersion (using a gamma distribution and log link function). In the global model, fixed variables are landscape, sequencing depth (scaled), field season, and sex, and random intercepts set for study site nested within landscape and for extraction batch. Models are ranked according to their Akaike information criterion adjusted for small sample sizes (AIC_C) from highest model support (lowest AIC_C value) to lowest model support (highest AIC_C value). The plus sign (+) indicates which variables are included in that specific candidate model. For each candidate model, the degrees of freedom (df), marginal and conditional coefficients of determination ($R^2_{GLMM(m)}$and $R^2_{GLMM(c)}$), log likelihood (logLik), difference in AIC_C to the AIC_C of the top ranked model (ΔAIC_C), and the Akaike weights ($AIC_C\omega$) are shown.

Supplementary Data 10: Model selection table of generalized linear mixed models and unweighted UniFrac distances to the study site centroids as a response variable to study differences in dispersion (using a gamma distribution and log link function). In the global model, fixed variables are landscape, sequencing depth (scaled), field season, and sex, and random intercepts set for study site nested within landscape and for extraction batch. Models are ranked according to their Akaike information criterion adjusted for small sample sizes (AIC_C) from highest model support (lowest AIC_C value) to lowest model support (highest AIC_C value). The plus sign (+) indicates which variables are included in that specific

candidate model. For each candidate model, the degrees of freedom (df), marginal and conditional coefficients of determination ($R^2_{GLMM(m)}$ and $R^2_{GLMM(c)}$), log likelihood (logLik), difference in AIC_C to the AIC_C of the top ranked model (ΔAIC_C), and the Akaike weights ($AIC_C\omega$) are shown.

Supplementary Data 11: Beta diversity distance to study site centroids generalized linear mixed models' model-averaged conditional parameter estimates and 95% confidence intervals. For each beta diversity distance metric (weighted and unweighted UniFrac), generalized linear mixed models were averaged with a cumulative AIC_C weight (AIC_C ω) of 0.95. Each model contained the fixed variables landscape, sequencing depth (scaled), field season, and sex, and random intercepts set for study site nested within landscape and for extraction batch. The explanatory variables listed are those that were retained in the averaged models. For each explanatory variable in each model, we report the parameter estimate (β) and upper and lower 95% confidence intervals (upper/lower 95% CI). Please note that because the models used here were built using a log link function, the parameter estimates and 95% confidence intervals are log transformed.

Supplementary Data 12: Model comparison of generalized linear mixed models and weighted and unweighted UniFrac distances to the study site centroids as a response variable with and without a spatial autocorrelation parameter. In each model, fixed variables are landscape, sequencing depth (scaled), field season, and sex, and random intercepts set for study site nested within landscape and for extraction batch. For each beta diversity metric, there is a model with and without an expontential spatial autocorrelation parameter. Within each beta diversity metric, models are ranked according to their Akaike information criterion adjusted for small sample sizes (AIC_C) from highest model support (lowest AIC_C value) to lowest model support (highest AIC_C value). The plus sign (+) indicates which models included the spatial autocorrelation parameter. For each model, the degrees of freedom (df), log likelihood (logLik), difference in AIC_C to the AIC_C of the top ranked model within an alpha diversity metric (ΔAIC_C), and the Akaike weights ($AIC_C\omega$) are shown.

Supplementary Data 13: Model selection table of generalized linear mixed models with a negative binomial distribution and observed number of ASVs as a response variable (and including host density as an explanatory variable). In the global model, fixed variables are landscape, sequencing depth (scaled), field season, sex, and *P. semispinosus* density (scaled; individuals per hectare) and random intercepts set for study site nested within landscape and for extraction batch. Models are ranked according to their Akaike information criterion adjusted for small sample sizes (AIC_C) from highest model support (lowest AIC_C value) to lowest model support (highest AIC_C value). The plus sign (+) indicates which variables are included in that specific candidate model. For each candidate model, the degrees of freedom (df), marginal and conditional coefficients of determination ($R^2_{GLMM(m)}$ and

$R^2_{GLMM(c)}$), log likelihood (logLik), difference in AIC_C to the AIC_C of the top ranked model (ΔAIC_C), and the Akaike weights ($AIC_C\omega$) are shown.

Supplementary Data 14: Model selection table of generalized linear mixed models with a gamma distribution and log link function and Shannon diversity as a response variable (and including host density as an explanatory variable). In the global model, fixed variables are landscape, sequencing depth (scaled), field season, and sex, and *P. semispinosus* density (scaled; individuals per hectare) and random intercepts set for study site nested within landscape and for extraction batch. Models are ranked according to their Akaike information criterion adjusted for small sample sizes (AIC_C) from highest model support (lowest AIC_C value) to lowest model support (highest AIC_C value). The plus sign (+) indicates which variables are included in that specific candidate model. For each candidate model, the degrees of freedom (df), marginal and conditional coefficients of determination ($R^2_{GLMM(m)}$ and $R^2_{GLMM(c)}$), log likelihood (logLik), difference in AIC_C to the AIC_C of the top ranked model (ΔAIC_C), and the Akaike weights ($AIC_C\omega$) are shown.

Supplementary Data 15: Model selection table of generalized linear mixed models with a gamma distribution and log link function and Faith's phylogenetic diversity as a response variable (and including host density as an explanatory variable). In the global model, fixed variables are landscape, sequencing depth (scaled), field season, sex, and *P. semispinosus* density (scaled; individuals per hectare) and random intercepts set for study site nested within landscape and for extraction batch. Models are ranked according to their Akaike information criterion adjusted for small sample sizes (AIC_C) from highest model support (lowest AIC_C value) to lowest model support (highest AIC_C value). The plus sign (+) indicates which variables are included in that specific candidate model. For each candidate model, the degrees of freedom (df), marginal and conditional coefficients of determination ($R^2_{GLMM(m)}$ and $R^2_{GLMM(c)}$), log likelihood (logLik), difference in AIC_C to the AIC_C of the top ranked model (ΔAIC_C), and the Akaike weights ($AIC_C\omega$) are shown.

Supplementary Data 16: Alpha diversity generalized linear mixed models' (including host density as an explanatory variable) model-averaged conditional parameter estimates and 95% confidence intervals. For each of the three alpha diversity metrics (observed number of ASVs, Shannon diversity and Faith's phylogenetic diversity), generalized linear mixed models were averaged with a cumulative AIC_C weight ($AIC_C\omega$) of 0.95. Each model contained the fixed variables landscape, sequencing depth (scaled), field season, sex, and *P. semispinosus* density (scaled; individuals per hectare), and random intercepts set for study site nested within landscape and for extraction batch. The explanatory variables listed are those that were retained in the averaged models. For each explanatory variable in each model, we report the parameter estimate (β) and upper and lower 95% confidence intervals (upper/lower 95% CI). Please note that because the models used here were built using a log link function, the parameter estimates and 95% confidence intervals are log transformed.

Supplementary Data 17: Model selection table of generalized linear models with weighted UniFrac distances as a response variable (and including host density as an explanatory variable) to study shifts in gut community composition using the output from the *adonis* function. In the global model, fixed variables are landscape, study site nested within landscape, sequencing depth, field season, sex, extraction batch, and *P. semispinosus* density (scaled; individuals per hectare). The nested variable study site is conformably passed through the *strata* argument. Models are ranked according to their Akaike information criterion adjusted for small sample sizes (AIC_C) from highest model support (lowest AIC_C value) to lowest model support (highest AIC_C value). The plus sign (+) indicates which variables are included in that specific candidate model. For each candidate model, the coefficient of determination (R^2), log likelihood (logLik), difference in AIC_C to the AIC_C of the top ranked model (ΔAIC_C), and the Akaike weights ($AIC_C\omega$) are shown.

Supplementary Data 18: Model selection table of generalized linear models with unweighted UniFrac distances as a response variable (and including host density as an explanatory variable) to study shifts in gut community composition using the output from the *adonis* function. In the global model, fixed variables are landscape, study site nested within landscape, sequencing depth, field season, sex, extraction batch, and *P. semispinosus* density (scaled; individuals per hectare). The nested variable study site is conformably passed through the *strata* argument. Models are ranked according to their Akaike information criterion adjusted for small sample sizes (AIC_C) from highest model support (lowest AIC_C value) to lowest model support (highest AIC_C value). The plus sign (+) indicates which variables are included in that specific candidate model. For each candidate model, the coefficient of determination (R^2), log likelihood (logLik), difference in AIC_C to the AIC_C of the top ranked model (ΔAIC_C), and the Akaike weights ($AIC_C\omega$) are shown.

Supplementary Data 19: Results from null hypothesis significance testing with weighted and unweighted UniFrac distances as response variables (and including host density as an explanatory variable) to study shifts in gut community composition using the *adonis* function. In the model, explanatory variables are (in order): study site nested within landscape, landscape, field season, sex, sequencing depth, extraction batch, and *P. semispinosus* density (scaled; individuals per hectare). The nested variable study site is conformably passed through the *strata* argument. For each explanatory variable, the degrees of freedom (df), F-values, coefficients of determination (R^2), and *p*-values for 9,999 permutations are shown.

Supplementary Data 20: Model selection table of generalized linear mixed models and weighted UniFrac distances to the study site centroids as a response variable to study differences in dispersion (using a gamma distribution and log link function and including

host density as an explanatory variable). In the global model, fixed variables are landscape, sequencing depth (scaled), field season, sex, and *P. semispinosus* density (scaled; individuals per hectare), and random intercepts set for study site nested within landscape and for extraction batch. Models are ranked according to their Akaike information criterion adjusted for small sample sizes (AIC_C) from highest model support (lowest AIC_C value) to lowest model support (highest AIC_C value). The plus sign (+) indicates which variables are included in that specific candidate model. For each candidate model, the degrees of freedom (df), marginal and conditional coefficients of determination ($R^2_{GLMM(m)}$ and $R^2_{GLMM(c)}$), log likelihood (logLik), difference in AIC_C to the AIC_C of the top ranked model (ΔAIC_C), and the Akaike weights ($AIC_C\omega$) are shown.

Supplementary Data 21: Model selection table of generalized linear mixed models and unweighted UniFrac distances to the study site centroids as a response variable to study differences in dispersion (using a gamma distribution and log link function and including host density as an explanatory variable). In the global model, fixed variables are landscape, sequencing depth (scaled), field season, sex, and *P. semispinosus* density (scaled; individuals per hectare), and random intercepts set for study site nested within landscape and for extraction batch. Models are ranked according to their Akaike information criterion adjusted for small sample sizes (AIC_C) from highest model support (lowest AIC_C value) to lowest model support (highest AIC_C value). The plus sign (+) indicates which variables are included in that specific candidate model. For each candidate model, the degrees of freedom (df), marginal and conditional coefficients of determination ($R^2_{GLMM(m)}$ and $R^2_{GLMM(c)}$), log likelihood (logLik), difference in AIC_C to the AIC_C of the top ranked model (ΔAIC_C), and the Akaike weights ($AIC_C\omega$) are shown.

Supplementary Data 22: Beta diversity distance to study site centroids generalized linear mixed models' (including host density as an explanatory variable) model-averaged conditional parameter estimates and 95% confidence intervals. For each beta diversity distance metric (weighted and unweighted UniFrac), generalized linear mixed models were averaged with a cumulative AIC_C weight ($AIC_C\omega$) of 0.95. Each model contained the fixed variables landscape, sequencing depth (scaled), field season, sex, and *P. semispinosus* density (scaled; individuals per hectare), and random intercepts set for study site nested within landscape and for extraction batch. The explanatory variables listed are those that were retained in the averaged models. For each explanatory variable in each model, we report the parameter estimate (β) and upper and lower 95% confidence intervals (upper/lower 95% CI). Please note that because the models used here were built using a log link function, the parameter estimates and 95% confidence intervals are log transformed.
