# Supplementary material for: Human encroachment into wildlife gut microbiomes
Source: Commun Biol. 2021 Jun 25;4:800. doi: 10.1038/s42003-021-02315-7 (PMC8233340; doi:10.1038/s42003-021-02315-7)
Supplement: Supplementary file 4 — Reporting Summary [file 42003_2021_2315_MOESM4_ESM.pdf]

nature research

Corresponding author(s): Gloria Fackelmann & Simone Sommer

Last updated by author(s): Jan 28, 2021

Reporting Summary

Nature Research wishes to improve the reproducibility of the work that we publish. This form provides structure for consistency and transparency in reporting. For further information on Nature Research policies, see our Editorial Policies and the Editorial Policy Checklist.

Statistics

For all statistical analyses, confirm that the following items are present in the figure legend, table legend, main text, or Methods section.

n/a	Confirmed	
☐	☒	The exact sample size (*n*) for each experimental group/condition, given as a discrete number and unit of measurement
☐	☒	A statement on whether measurements were taken from distinct samples or whether the same sample was measured repeatedly
☒	☐	The statistical test(s) used AND whether they are one- or two-sided<br>*Only common tests should be described solely by name; describe more complex techniques in the Methods section.*
☐	☒	A description of all covariates tested
☐	☒	A description of any assumptions or corrections, such as tests of normality and adjustment for multiple comparisons
☐	☒	A full description of the statistical parameters including central tendency (e.g. means) or other basic estimates (e.g. regression coefficient) AND variation (e.g. standard deviation) or associated estimates of uncertainty (e.g. confidence intervals)
☒	☐	For null hypothesis testing, the test statistic (e.g. *F*, *t*, *r*) with confidence intervals, effect sizes, degrees of freedom and *P* value noted<br>*Give P values as exact values whenever suitable.*
☒	☐	For Bayesian analysis, information on the choice of priors and Markov chain Monte Carlo settings
☒	☐	For hierarchical and complex designs, identification of the appropriate level for tests and full reporting of outcomes
☐	☒	Estimates of effect sizes (e.g. Cohen's *d*, Pearson's *r*), indicating how they were calculated

Our web collection on statistics for biologists contains articles on many of the points above.

Software and code

Policy information about availability of computer code

Data collection	No software was used.
Data analysis	DADA2 QIIME 2 plugin (version 2018.6.0) (Bolyen et al., 2019; Callahan et al., 2016).<br>q2-composition QIIME 2 plugin (version 2018.6.0) (Bolyen et al., 2019).<br>FastTree (version 2.1.10 Double precision) (Price et al., 2010).<br>Dendroscope (version 3.5.10) (Huson & Scornavacca, 2012).<br>R (version 3.6.0) (R Core Team, 2017).<br>R phyloseq package (McMurdie & Holmes, 2013).<br>R vegan package (Oksanen et al., 2019).<br>R lme4 package (Bates et al., 2015).<br>R ANCOM package (Mandal et al., 2015).

For manuscripts utilizing custom algorithms or software that are central to the research but not yet described in published literature, software must be made available to editors and reviewers. We strongly encourage code deposition in a community repository (e.g. GitHub). See the Nature Research guidelines for submitting code & software for further information.

Data

Policy information about availability of data

All manuscripts must include a data availability statement. This statement should provide the following information, where applicable:

- Accession codes, unique identifiers, or web links for publicly available datasets
- A list of figures that have associated raw data
- A description of any restrictions on data availability

All data and accession codes will be available before publication.

Field-specific reporting

Please select the one below that is the best fit for your research. If you are not sure, read the appropriate sections before making your selection.

☐ Life sciences ☐ Behavioural & social sciences ☒ Ecological, evolutionary & environmental sciences

For a reference copy of the document with all sections, see nature.com/documents/nr-reporting-summary-flat.pdf

Ecological, evolutionary & environmental sciences study design

All studies must disclose on these points even when the disclosure is negative.

Study description	Here, we studied the gut microbial composition of 384 individuals of a generalist rodent species, Tome's spiny rat Proechimys semispinosus, in seventeen study sites in three tropical landscapes differing in their degree of anthropogenic environmental change in Panama, Central America. The three landscapes encompassed: protected continuous tropical forests and protected forested islands in the Panama Canal that allow us to study the effects of fragmentation on its own – both landscapes have no anthropogenic disturbance - and nearby unprotected forested fragments embedded in an agricultural matrix that are subjected to anthropogenic stressors. By comparing protected, fragmented sites to heavily human-disturbed, fragmented sites, our unique study design allowed us to pick apart the effects of habitat fragmentation (i.e. habitat reduction and isolation) from those of anthropogenic disturbance (i.e. contact with livestock and humans within an agricultural matrix). Within each landscape, rats were sampled in at least five different study sites (sites C1-C5 nC = 103, A1-A6 nC = 145, and I1-I6 nI = 136, ntotal = 384 P. semispinosus individuals).
Research sample	A group of individually marked Tome's spiny rats (P. semispinosus).
Sampling strategy	At each of the 17 study sites, trapping stations were set at 20 m intervals along parallel trapping lines, so that each study site harbored a maximum of 100 evenly spaced trapping stations, whereby each trapping station consisted of three traps. This was done to maximize the number of individuals which could be sampled to be able to statistically detect any potentially small landscape effects.
Data collection	Fecal samples were collected non-invasively by natural defecation during field work in Panama. All animals were released at the site of capture immediately after sampling. The microbiome data were generated using an Illumina MiSeq sequencing platform at the Sommer Lab at Ulm University in Ulm, Germany.
Timing and spatial scale	Each of the 17 study sites were sampled once per field season across five consecutive nights and this study encompassed three field seasons: October 2013 to May 2014, October 2014 to May 2015, and September 2016 to April 2017. Sampling took place three times in the same period of the year outside the reproductive season of P. semispinosus. Study sites were chosen by ensuring that the vegetation cover was similar among all sites.
Data exclusions	The only analysis that required some data exclusions was the PERMDISP2 analysis. This is because PERMDSIP2 is sensitive to variations in sample sizes between treatments (Anderson, 2006; Anderson & Walsh, 2013). Therefore, we performed this test using only study sites for which we had data for 15 or more individuals (C1-C4 nC= 89, A2-A3 nA = 107, and I1, I3-I6 nI = 126, ntotal = 322 individuals).
Reproducibility	All attempts to repeat the experiment were successful.
Randomization	Individual Tome's spiny rats were allocated into groups based on their study site.
Blinding	The field team in Panama that collected the fecal samples was not involved in the microbiome analysis in Ulm, Germany.

Did the study involve field work? ☒ Yes ☐ No

Field work, collection and transport

Field conditions	This study was carried out in the tropical rainforest in Panama and field assistants were based at the Smithsonian Tropical Research Institute on Barro Colorado Island, which provided all necessary logistic and safety measures.
Location	Panama, Central America.<br>Study site latitudes and longitudes:<br>A1,9.11181000,-79.87603000

A2,9.26416200,-79.65658795
A3,9.27780600,-79.65625388
A4,9.25706200,-79.65603239
A5,9.25844639,-79.70803056
A6,9.24847556,-79.70305778
C1,9.20456900,-79.82975277
C2,9.11403500,-79.86038545
C3,9.16208000,-79.80084416
C4,9.15268400,-79.88575228
C5,9.11700100,-79.83732775
I1,9.20788800,-79.90755130
I2,9.14539700,-79.85723736
I3,9.20497900,-79.84758110
I4,9.21126800,-79.89184037
I5,9.13756400,-79.83414904
I6,9.17872800,-79.84810283

Access & import/export

This study was carried out within the framework of the DFG (German Science Foundation) Priority Program SPP 1596/2 Ecology and Species Barriers in Emerging Infectious Diseases (SO 428/9-1, 9-2, with full ethical approval according to the Smithsonian IACUC protocol 2013-0401-2016-A1-A7 and 2016-0627-2019-A1-A2). Permission to export samples to Germany was granted by the Panamanian government (SE/A-21-14, SE/A-69-14, SEX/A-22-15, SEX/A-24-17, SEX/A-120-16, and SEX/A-52-17).

Disturbance

This project was approved by the Smithsonian Tropical Research Institute and the Panamanian Government. No animal was killed for the purpose of this study.

Reporting for specific materials, systems and methods

We require information from authors about some types of materials, experimental systems and methods used in many studies. Here, indicate whether each material, system or method listed is relevant to your study. If you are not sure if a list item applies to your research, read the appropriate section before selecting a response.

Materials & experimental systems

n/a	Involved in the study	
☒	☐	Antibodies
☒	☐	Eukaryotic cell lines
☒	☐	Palaeontology and archaeology
☐	☒	Animals and other organisms
☒	☐	Human research participants
☒	☐	Clinical data
☒	☐	Dual use research of concern

Methods

n/a	Involved in the study	
☒	☐	ChIP-seq
☒	☐	Flow cytometry
☒	☐	MRI-based neuroimaging

Animals and other organisms

Policy information about studies involving animals; ARRIVE guidelines recommended for reporting animal research

Laboratory animals

This study did not involve laboratory animals.

Wild animals

Proechimys semispinosus (Tome's spiny rat) individuals were live-trapped at each study site once per field season (three field seasons total: October 2013 to May 2014, October 2014 to May 2015, and September 2016 to April 2017) across five consecutive nights. At each study site, trapping stations were set at 20 m intervals along parallel trapping lines, so that each study site harbored a maximum of 100 evenly spaced trapping stations. Each trapping station contained three individual traps: one Tomahawk trap (15.2 cm × 15.2 cm × 48.3 cm, livetrap.com) placed on the ground and two Sherman traps (10.2 cm × 11.4 cm × 38.1 cm, shermantraps. com), one placed on the ground and one attached to a liana or tree branch at a height of 0.5–2.5 m whenever possible. To identify recaptures and prevent pseudoreplicates, each individual was marked using animal marking sticks (Raidex GmbH, Dettingen/Erms, Germany). Individuals were immediately released at the site of capture.

Field-collected samples

In the field, fecal samples were stored in Eppendorf tubes containing RNAlater and transferred to -20 °C upon daily return back to the field station.

Ethics oversight

This study was carried out within the framework of the DFG (German Science Foundation) Priority Program SPP 1596/2 Ecology and Species Barriers in Emerging Infectious Diseases (SO 428/9-1, 9-2, with full ethical approval according to the Smithsonian IACUC protocol 2013-0401-2016-A1-A7 and 2016-0627-2019-A1-A2). Permission to export samples to Germany was granted by the Panamanian government (SE/A-21-14, SE/A-69-14, SEX/A-22-15, SEX/A-24-17, SEX/A-120-16, and SEX/A-52-17).

Note that full information on the approval of the study protocol must also be provided in the manuscript.
